# Genome-scale analysis of *Acetobacterium bakii* reveals the cold adaptation of psychrotolerant acetogens by post-transcriptional regulation

**DOI:** 10.1261/rna.068239.118

**Published:** 2018-12

**Authors:** Jongoh Shin, Yoseb Song, Sangrak Jin, Jung-Kul Lee, Dong Rip Kim, Sun Chang Kim, Suhyung Cho, Byung-Kwan Cho

**Affiliations:** 1 Department of Biological Sciences and KI for the BioCentury, Korea Advanced Institute of Science and Technology, Daejeon 34141, Republic of Korea; 2 Department of Chemical Engineering, Konkuk University, Seoul 05029, Republic of Korea; 3 Department of Mechanical Engineering, Hanyang University, Seoul 04763, Republic of Korea; 4 Intelligent Synthetic Biology Center, Daejeon 34141, Republic of Korea

**Keywords:** post-transcriptional regulation, psychrotolerant acetogen, *Acetobacterium bakii*, cold-adaptive acetogenesis

## Abstract

Acetogens synthesize acetyl-CoA via CO_2_ or CO fixation, producing organic compounds. Despite their ecological and industrial importance, their transcriptional and post-transcriptional regulation has not been systematically studied. With completion of the genome sequence of *Acetobacterium bakii* (4.28-Mb), we measured changes in the transcriptome of this psychrotolerant acetogen in response to temperature variations under autotrophic and heterotrophic growth conditions. Unexpectedly, acetogenesis genes were highly up-regulated at low temperatures under heterotrophic, as well as autotrophic, growth conditions. To mechanistically understand the transcriptional regulation of acetogenesis genes via changes in RNA secondary structures of 5′-untranslated regions (5′-UTR), the primary transcriptome was experimentally determined, and 1379 transcription start sites (TSS) and 1100 5′-UTR were found. Interestingly, acetogenesis genes contained longer 5′-UTR with lower RNA-folding free energy than other genes, revealing that the 5′-UTRs control the RNA abundance of the acetogenesis genes under low temperature conditions. Our findings suggest that post-transcriptional regulation via RNA conformational changes of 5′-UTRs is necessary for cold-adaptive acetogenesis.

## INTRODUCTION

Microbial conversion of single carbon (C_1_) compounds, such as CO and CO_2_, to biofuels and commodity chemicals, has been considered as one of the sustainable routes for the replacement of fossil resources (Henstra et al. 2007; Bengelsdorf et al. 2013; Latif et al. 2014). In particular, acetogens are attractive biocatalysts, since they can grow autotrophically with CO_2_ as the sole carbon source and H_2_ as the electron donor, producing acetate as a primary end product (Drake 1995). This unique metabolism in acetogens is mediated by the reductive acetyl-CoA pathway referred to as the Wood–Ljungdahl pathway (WLP), which provides a highly efficient system for converting CO and CO_2_ to various biochemicals including acetate, ethanol, 2,3-butanediol, butyrate, and butanol (Schiel-Bengelsdorf and Dürre 2012).

Acetogens are physiologically defined as a group of anaerobic bacteria that synthesize acetyl-CoA as a central metabolic intermediate from chemolithoautotrophic substrates (Drake 1995). Acetogens comprise at least 23 different genera (Drake et al. 2006) and their growth environments range from anoxic freshwater, soils, marine sediments, and sewage sludge to the gastrointestinal tracts of insects and animals (Drake et al. 2006). Thus, systematic understanding of their genetic backgrounds, as well as of transcriptional and translational regulation in response to environmental factors, is a prerequisite for the full exploitation of their biosynthetic potential. One example is the anaerobic growth of acetogens at low temperatures, of particular interest in view of its ecological significance (Nozhevnikova et al. 1994). Several cold-active acetogens were isolated from cold environments (Nozhevnikova et al. 1994, 2001; Kotsyurbenko et al. 1995, 2001; Simankova et al. 2000) including perennially ice-covered sediments (Sattley and Madigan 2007). They are all affiliated with the genus *Acetobacterium*, reflecting their phylogenetic proximity. The isolated bacteria are all psychrotolerant and proliferate at temperatures as low as 1°C. Although both mesophilic and thermophilic acetogens have been extensively studied, the mechanisms of cold adaptation and the regulation of acetogenesis, including the WLP and the energy conservation systems of psychrotolerant acetogens, remain elusive.

In transcriptional and post-transcriptional regulation, DNA sequences such as principal promoter elements and 5′-untranslated regions (5′-UTRs) play a critical role as genome-embedded *cis*-acting determinants (Jeong et al. 2016). The identification of transcription start sites (TSSs) via differential RNA sequencing (dRNA-seq) has facilitated the definition of bacterial operons and regulatory sequences (Sharma et al. 2010; Sharma and Vogel 2014). The latter approach, combined with RNA sequencing (RNA-seq) for the study of gene expression, now allows for quantitative analysis of transcriptional and translational regulation in various species with unprecedented detail (Sharma and Vogel 2014; Jeong et al. 2017). Also, the precise mapping of TSSs enables the definition of the sequence and structure of the mRNA 5′ ends, thus providing insights into mRNA stability and translational efficiency (Jeong et al. 2016). Thus, we describe a systematic approach toward the integration of genome, primary transcriptome, and transcriptome data from a psychrotolerant acetogen, *Acetobacterium bakii* DSM 8239, originally isolated from a cold environment (<6°C) (Kotsyurbenko et al. 1995) and displaying a higher growth rate at 1°C than *Acetobacterium paludosum* and *Acetobacterium fimetarium* (Kotsyurbenko et al. 1995). We completed the *A. bakii* genome sequence and identified unique expression patterns of acetogenesis genes under optimal and low temperature growth conditions. We also determined the genome-wide location of TSSs to investigate the role of *cis*-acting elements including 5′-UTRs, promoter elements, and Shine-Dalgarno (SD) sequences. Our data suggest that the RNA regulatory elements embedded in the 5′-UTR orchestrate the gene expression changes underlying adaptation to a cold environment.

## RESULTS

### Completion of the genome sequence of the psychrotolerant bacterium *A. bakii*

To complete the *A. bakii* genome sequence, we used single-molecule real-time (SMRT) sequencing, yielding 108,817 quality-trimmed long reads (6297 bp of average length). The genome was assembled using a hierarchical genome assembly process (HGAP v2.3.0), the SSPACE scaffolding algorithm (Hunt et al. 2014) with Illumina mate-pair sequencing data, and a PCR-based primer walking method. The genome was polished using two types of short high-fidelity sequence reads obtained from whole-genome paired-end sequencing and strand-specific RNA sequencing (RNA-seq). One scaffold (4.28-Mb) and one short contig (35-kb) were finally assembled as a chromosome and a plasmid DNA, respectively (Supplemental Table S1). However, the 35-kb contig could not be assembled as a circular DNA, suggesting that it was an unfinished product resulting from incomplete assembly. A total of 112 nt conflicts between Illumina short read sequences and the assembled genome sequence were corrected by the Illumina short read sequences (Supplemental Table S2). The 4.28-Mb genome, with a GC content of 41.3%, was annotated for 3973 coding genes (CDS), 22 ribosomal RNAs (rRNA), and 69 transfer RNAs (tRNA) genes. Compared to the previous draft genome sequence (Hwang et al. 2015), an additional 183,963 bps and 280 genes were identified including large repeats and structural variations, such as rRNA genes (Supplemental Table S1). Importantly, the methyl-branch of the WLP, including a formyl-tetrahydrofolate (THF) synthetase (ABAKI_ c24790) and a V-type ATP synthase gene cluster (ABAKI_ c03210 – ABAKI_c03290), were newly uncovered in the completed genome.

**FIGURE 1.**
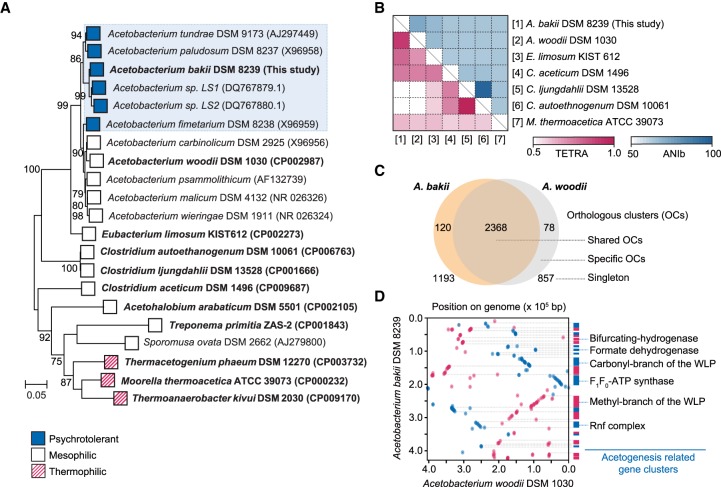
Comparative genome analysis. (*A*) Phylogenetic tree of the selected 21 acetogens including psychrotolerant, mesophilic, and thermophilic acetogens. Bootstrap values are shown at nodes and low bootstrap values (<70) were clipped based on 1000 trials. The maximum-likelihood phylogenetic tree was constructed in MEGA v6.06 (Tamura et al. 2013). The 16S rRNA gene sequences, obtained from genome sequence data, are shown in bold. The accession numbers for each sequence are shown after the strain name. (*B*) Pairwise comparison of the average nucleotide identity based on BLAST+ (ANIb) and tetranucleotide composition (TETRA) values among the available genomes of seven acetogen species analyzed via JSpeciesWS (Richter et al. 2016). Each value is shown as a row and a column with the order of species being the same on both axes. (*C*) Venn diagram of orthologous gene cluster between *A. bakii* and *A. woodii*. (*D*) An alignment plot between the *A. bakii* scaffold 1 and *A. woodii* reference genome. This plot was generated using NUCmer version 3.1 (Kurtz et al. 2004) from the MUMMER package. The syntenic blocks, representing conserved segments, were generated using Mauve alignment with default parameters (Darling et al. 2010). All the syntenic blocks were highlighted with two distinguishable colors in this dot plot.

The genomes of several model acetogens, such as *Acetobacterium woodii* (Poehlein et al. 2012), *Clostridium ljungdahlii* (Köpke et al. 2010), *Clostridium autoethanogenum* (Brown et al. 2014), and *Moorella thermoacetica* (Pierce et al. 2008), have been reported. Despite their genetic diversity, acetogens share highly conserved gene clusters for autotrophic growth, such as the WLP, central metabolic pathways, and cofactor biosynthetic pathways (Poehlein et al. 2015; Shin et al. 2016). Pairwise genome comparison showed that *A. bakii* was most closely related to *A. woodii* (Fig. 1A), as confirmed by a tetranucleotide frequency correlation coefficient of 0.92 and an average nucleotide identity of 73.6%, in agreement with the 16S rDNA-based phylogenetic analysis (Fig. 1B). Thus, we compared the *A. bakii* genome with the *A. woodii* genome to determine distinctive genetic features. Overall, 2368 orthologous clusters (2483 genes, 62.5%) were identified (Fig. 1C). Furthermore, the high level of synteny, as well as the similarity ranging from 78.9% to 100% between *A. bakii* and *A. woodii,* were reflected in the identification of 221 specific alignments, encoding key enzymes of the WLP and the energy conservation system (Fig. 1D). Taken together, the genome sequence provides not only the complete acetogenesis pathway for autotrophic growth but also the basis for a comprehensive view of the genetic architecture of the *A. bakii* genome.

### The Wood–Ljungdahl pathway and the energy conservation system in *A. bakii*

**FIGURE 2.**
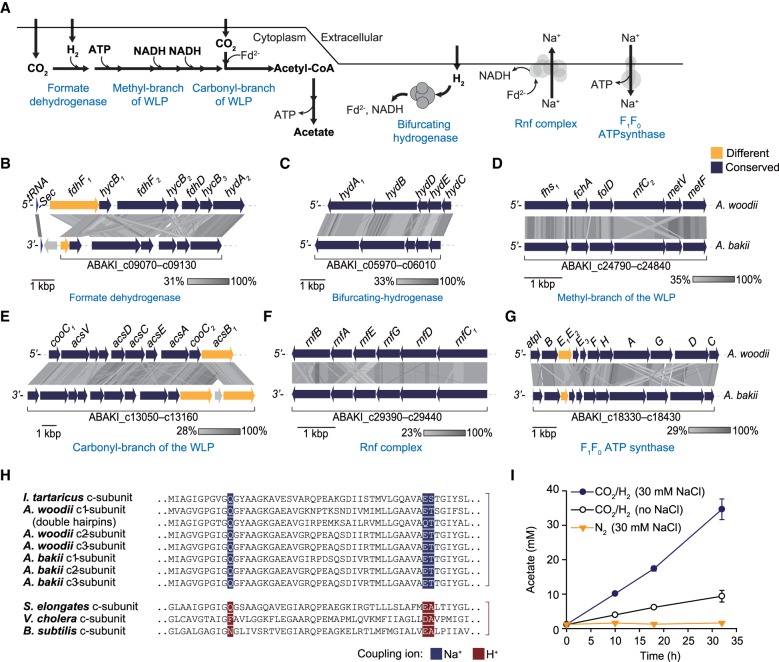
The WLP of *A. bakii*. (*A*) Schematic representation of the putative acetogenesis in *A. bakii*. (*B*–*G*) Synteny analysis of major gene clusters involved in acetogenesis between *A. bakii* and *A. woodii*. Comparisons were performed using tBLASTx within Easyfig (Sullivan et al. 2011), and each arrow indicates a gene. Genetic conservation is represented by a color. (*H*) Comparison of the sequences of the *c*-subunits of ATP synthase. These *c*-subunits assembled into homo- or heteromeric *c*-rings and play a direct role in ion translocation across the membrane. To determine the Na^+^/H^+^ binding motif, the *c*-subunit sequences of F_1_F_0_-type ATP synthase complex were aligned. The Na^+^/H^+^ binding motif, either predicted (*A. bakii*) or known (other strains), is highlighted in color in each case. Used *c*-subunit sequences were obtained by the following accession numbers or locus_tags: *Ilyobacter tartaricus* (Q8KRV3), *Acetobacterium woodii* (Awo_c02160 – c02180), *Acetobacterium bakii* (ABAKI_c18390–c18410), *Synechococcus elongates* (YP_399351), *Vibrio cholera* (Q9KNH0), and *Bacillus subtilis* (P37815). (*I*) Resting cell assay was performed with or without 30 mM NaCl. The whole cell of *A. bakii* (0.25 mg/mL) was incubated with 50 mM imidazole, 20 mM MgSO_4_, 20 mM KCl, 4.4 µM resazurin, 4 mM DTE at pH 7.0. The gaseous atmosphere was H_2_–CO_2_ (80:20, v/v, 200 kPa) or N_2_ (200 kPa). All values are means obtained from two independent experiments.

Acetogenesis is initiated by the reduction of CO_2_ to formate via a reversible formate dehydrogenase (FDH) activity (Fig. 2A). A hydrogen-dependent CO_2_ reductase (*HDCR*) gene cluster (ABAKI_c09070 – ABAKI_c09130), consisting of genes substantially similar (87.7%–97.3% similarity at the nucleotide level) to those of *A. woodii*, included FDH (*fdhF1* or *fdhF2*), [FeFe]-hydrogenase (*hydA2*), and three hydrogenase Fe-S subunits (*hycB1*, *hycB2*, and *hycB3*) (Fig. 2B,C). Unlike the gene cluster in the *A. woodii* genome, a gene encoding selenium-free FDH (ABAKI_ c09130) was truncated and a molybdopterin-guanine dinucleotide biosynthesis protein (ABAKI_c09140) was located adjacent to the FDH cluster in the *A. bakii* genome.

In contrast to the single gene cluster found in the genus *Clostridium*, gene clusters encoding the methyl and the carbonyl branches of the WLP were separately located in the *A. bakii* genome (Fig. 2D,E). The enzymes of the methyl branch reduce CO_2_ to formate and subsequently convert formate to methyl-THF. These enzymes are formyl-tetrahydrofolate (THF) synthase (ABAKI_c24790), formyl-THF cyclohydrolase (ABAKI_c24800), methylene-THF dehydrogenase (ABAKI_c24810), methylene-THF reductase (ABAKI_c24840 for large subunit and ABAKI_c24830 for small subunit), and an additional electron transport complex subunit (ABAKI_c24820). In the carbonyl branch, acetyl-CoA is generated from methyl-CoFeSP and additional CO_2_ by a methyltransferase and a bifunctional CO dehydrogenase/Acetyl-CoA synthase (CODH/ACS). The CODH/ACS complex was encoded by *acsA* and *acsB* (ABAKI_c13090, ABAKI_c13050, and ABAKI_c13070), and the carbonyl branch was completed by a CODH maturation protein (ABAKI_c13160), corrinoid iron–sulfur protein subunits (ABAKI_c13110 and ABAKI_c13120), a methyltransferase (ABAKI_c13100), and a corrinoid activation and regeneration protein (ABAKI_c13150). Unlike *A. woodii*, an additional *acsB* (ABAKI_c13050), encoding an acetyl-CoA synthase and a hypothetical protein gene, were also clustered in the same region.

Energy conservation, in addition to ATP synthesis via acetate kinase, is required for the lithoautotrophic growth of acetogens (Schuchmann and Müller 2014). The energy conservation system of *A. bakii*, which is highly similar to that of *A. woodii*, consists of a bifurcating hydrogenase (ABAKI_c05970 – ABAKI_c06010), an Rnf complex (ABAKI_c29390 – ABAKI_c29440), and an F_1_F_0_ ATP synthase (ABAKI_c18330 – ABAKI_c18430). Hydrogen is oxidized by bifurcating hydrogenases, which generate equal moles of reduced ferredoxin and NADH via electron bifurcation (Fig. 2C). The reduced ferredoxin generates a sodium ion gradient and results in NADH production by the Rnf complex (Fig. 2F), after which the membrane-bound F_1_F_0_ ATP synthase drives ATP synthesis using the sodium ion gradient (Fig. 2G). This result supports the notion that the bioenergetic processes of *A. bakii* are identical to those of *A. woodii*. Sequence alignment analysis of the Na^+^- and H^+^-dependent *c* subunits of several F_1_F_0_ ATP synthase complexes indicates that *A. bakii* contains the conserved Na^+^ binding motif in the *c* subunit of F_1_F_0_ ATPase (Fig. 2H). Furthermore, the resting cell assays showed that *A. bakii* uses Na^+^ as a coupling ion during acetogenesis. In the absence of NaCl, the final concentration of acetate was about 9.4 mM, whereas 34.6 mM acetate was produced in the presence of 30 mM NaCl (Fig. 2I). Taken together, the comparative genome analysis indicates that the fundamental genetic components of acetogenesis are highly similar between *A. bakii* and *A. woodii*.

### Transcriptome changes under different growth conditions

**FIGURE 3.**
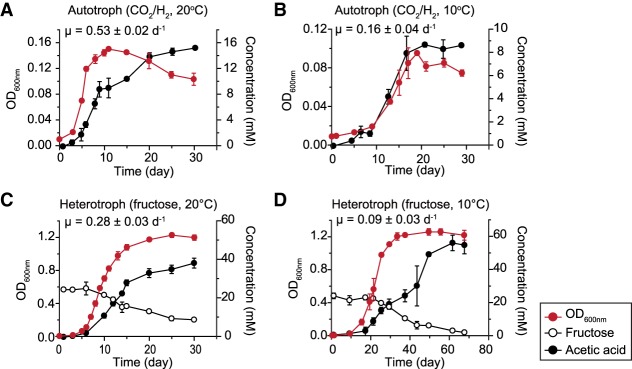
Growth and metabolite profiles *of A. bakii* DSM 8239. Growth curves and metabolite concentrations were measured under (*A*) 20°C autotrophic, (*B*) 10°C autotrophic, (C) 20°C heterotrophic, and (*D*) 10°C heterotrophic growth conditions. The data are presented as the mean of three different biological replicates ±SEM.

Genome annotation followed by comparative genome analysis identified the key genes required for chemolithotrophic acetogenic CO_2_/H_2_ utilization in *A. bakii*. To confirm the acetogenesis activity of *A. bakii*, cell growth was monitored under autotrophic (A) and heterotrophic (H) conditions at low (10°C) and high (20°C) temperatures (hereafter designated as A10, A20, H10, and H20). The specific growth rates (µ) were 0.53 ± 0.02 d^−1^ at 20°C and 0.16 ± 0.04 d^−1^ at 10°C under autotrophic conditions (Fig. 3A,B), whereas they were 0.28 ± 0.03 d^−1^ at 20°C and 0.09 ± 0.03 d^−1^ at 10°C under heterotrophic conditions (Fig. 3C,D). The production rate of acetate, the main product of acetogenesis, was dependent on cell growth. These results demonstrated that the psychrotolerant acetogen *A. bakii* displays autotrophic growth in the presence of CO_2_ and H_2_ at low temperature (Kotsyurbenko et al. 2001).

To measure the transcriptomic changes induced by different growth conditions, we performed RNA-seq experiments with cultures exponentially grown under A10, A20, H10, and H20 conditions. A total of 8.7–23.0 million sequence reads were mapped to the genome (Supplemental Table S3) with at least 100-fold genome-wide coverage and high strand-specificity (Supplemental Fig. S1A,B). Hierarchical clustering and principal component analysis of the sequencing results demonstrated a significant and reproducible difference in gene expression between the bacteria exposed to the four growth conditions (Supplemental Fig. S1C–E). RNA-seq data were normalized by DEseq2 (Love et al. 2014) to identify the differentially expressed genes (DEG). The expression levels of selected genes, encoding factors involved in WLP and energy conservation, were independently validated by quantitative reverse transcription PCR (qRT-PCR) analysis (Supplemental Fig. S1F). RNA-seq and qRT-PCR results were highly correlated (Pearson correlation coefficients >0.91), supporting the validity and reproducibility of the RNA-seq results.

**FIGURE 4.**
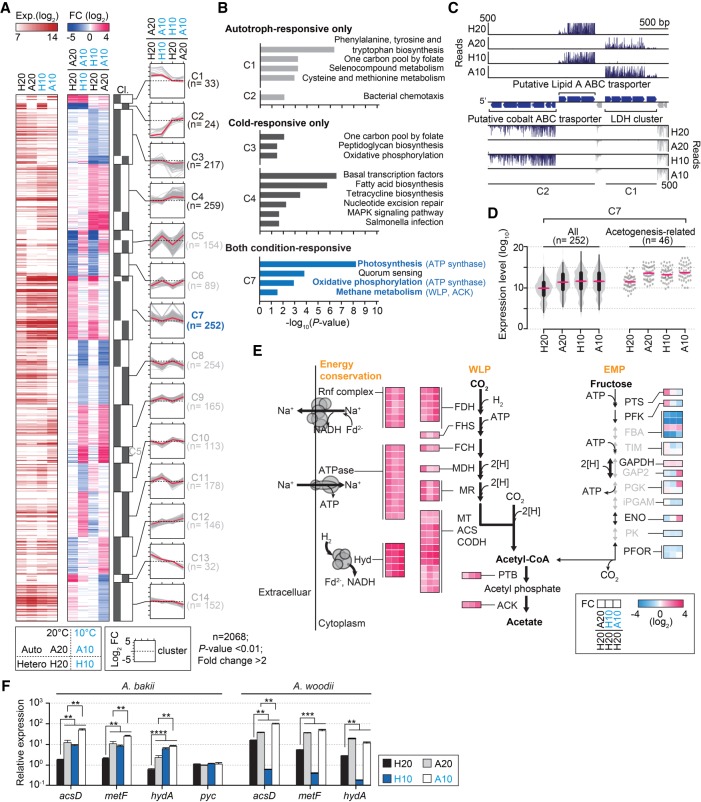
Transcriptome perturbations induced by the autotrophic and cold condition. (*A*) The autotroph-responsive only (C1 and C2), temperature-responsive only (C3 and C4), combined condition-responsive (C6, C7, C8, and C9), and particular condition-specific (C5, C10, C11, C12, C13, and C14) differential gene expression patterns were all combined and are shown as a heat map. The heat map shows log_2_ expression values (“Exp.”) and log_2_ fold-changes (“FC”) at 20°C heterotrophic (“H20”), 20°C autotrophic (“A20”), 10°C heterotrophic (“H10”), and 10°C autotrophic (“A10”) conditions. Black bars represent clustered columns resulting from *K*-mean clustering. Two thousand sixty-eight DEG are listed in Supplemental Table S4. C1 and C2 were highly up-regulated or down-regulated under autotrophic conditions, respectively; C3 and C4 were highly down-regulated or up-regulated at low temperature, respectively; C5 showed down-regulation of gene expression under A20, but their gene expression was inversely altered by cold temperature; the expression of C6 was down-regulated either by autotrophic or cold temperature conditions; the expression of C7 was up-regulated both by the autotrophic and cold temperature conditions; C8 and C9 were found to be highly down-regulated and up-regulated by A10, respectively; C10 and C11 were found to be highly up-regulated and down-regulated by H10, respectively; C12 and C13–C14 were found to be highly down-regulated or up-regulated by A20, respectively. Clustering was performed based on gene expression-fold change. Twenty-one clusters were assigned to 14 clusters manually. Gray and red lines indicate log_2_ (fold-changes) and median of each group, respectively. See also Supplemental Table S4 for all transcript abundances. (*B*) Enriched KEGG pathways of the C1, C2, C3, C4, and C7 clusters. Bonferroni-corrected *P* < 0.05 was considered as the significance cutoff. KEGG pathways, including acetogenesis-related genes, are highlighted in bold blue. For clarity of illustration, KEGG enrichment significance is shown as −log_10_ (*P*-value). See also Supplemental Figure S2. (*C*) An example of RNA-seq profiles for the genes in the A group. RNA-seq reads of both strands are shown. The putative cobalt ABC transporter cluster (ABAKI_c2420–c24260), a putative lipid A ABC transporter (ABAKI_c24270–c24290), and the bifurcating lactate dehydrogenase gene cluster (ABAKI_c24310–c24350) are highlighted in indigo blue. (*D*) A violin plot shows the expression distribution for the genes in the C7 group. See also Supplemental Figure S3. (*E*) DEG involved in acetogenesis, as well as in the glycolysis and gluconeogenesis pathways. Low temperature triggers the expression of acetogenesis-related genes. Bold and gray arrows indicate positive and negative regulation, respectively, under the cold-heterotrophic growth condition (H10). The heat map shows log_2_ fold-changes. See also Supplemental Table S5. (*F*) Quantitative RT-PCR data of *acsD* (from the carbonyl branch of the WLP), *metF* (from the methyl branch of the WLP), *hydA* (from the FDH operon), and *pyc* genes. The graph shows fold-changes in the transcript abundance of *A. bakii* and *A. woodii* for the four different growth conditions. Each value is the mean of three replicates in independently repeated experiments, and error bars show ±SEM. The *secA* gene was used as the reference. (**) *P* ≤ 0.01 > 0.001; (***) *P* < 0.001 (Student's unpaired *t*-test).

More than 3656 genes showed a normalized expression value of 10 or higher across all growth conditions, reflecting the transcription of 88% of the total annotated genes under at least one condition. Among them, 2068 genes showed significant changes in expression levels (fold change >2, *P*-value <0.01) under two or more conditions (Supplemental Table S4). The DEGs were then analyzed by performing an unsupervised *K*-mean clustering analysis, resulting in 21 clusters on the basis of the error sum of squares (SSE) analysis (Supplemental Fig. S1G). The genes belonging to these 21 differentially modulated clusters were further grouped into 14 clusters based on their growth condition-specific expression patterns (Fig. 4A). The genes in each group were also visualized on the Kyoto Encyclopedia of Genes and Genomes (KEGG) pathway (Fig. 4B; Supplemental Fig. S2). Interestingly, only 33 and 24 genes, belonging to the C1 and C2 clusters, were exclusively up-regulated under autotrophic and heterotrophic growth conditions, respectively (Fig. 4A; Supplemental Table S4). For example, consistent with the substrate-dependent regulation of the lactate dehydrogenase (LDH) operon previously reported for *A. woodii* (Weghoff et al. 2015), we found that the gene expression of the LDH gene in the C1 cluster was specifically repressed under heterotrophic growth conditions (Fig. 4C). In contrast, the expression of a putative lipid A ABC transport system (ABAKI_c24270 – c24290), a putative cobalt ABC transport system (ABAKI_c24200 – c24260), chemotaxis proteins (ABAKI_c30360 – c30400), and the ferrous iron transport protein gene clusters (ABAKI_c09680 – c09710) in the C2 cluster was highly and exclusively down-regulated under autotrophic conditions (Fig. 4B,C).

Temperature-dependent gene expression patterns were found in the C3 and C4 clusters. Despite that no common features were found, to date, among the cold-adapted prokaryotic genomes (Bowman 2008), cold-dependent regulation of gene expression has been largely reported, such as an increasing proportion of unsaturated fatty acids to maintain the fluidity of cellular membranes (Barria et al. 2013), a sensor histidine kinase as a cold sensor (Aguilar et al. 2001), and proteins preserving RNA secondary structures at low temperature (Barria et al. 2013), including Dead-box RNA helicase and cold shock proteins (CSP). Notably, we observed that the expression level of genes of the lipid biosynthesis pathway (ABAKI_c35860 – c35970), cold shock protein CspL (ABAKI_c09820 and ABAKI_c26430), and Dead-box helicase (ABAKI_c00160 – c00180, c00530, and c01100) was dramatically up-regulated under cold growth conditions, regardless of the carbon sources (Fig. 4B; Supplemental Table S4).

### Up-regulation of acetogenesis genes at low temperature

The mRNA transcripts of the WLP and hydrogenase-encoding genes in *C. autoethanogenum* (Marcellin et al. 2016), *C. ljungdahlii* (Tan et al. 2013), and *A. woodii* (Poehlein et al. 2012) were reported to be highly abundant under autotrophic conditions. Thus, it was predicted that most of the acetogenesis genes are activated by autotrophic conditions only, irrespective of growth temperatures, such as genes in the cluster C1. However, the cluster C7 contains most of the acetogenesis genes, including the F_1_F_0_ATP synthase operon, the Rnf complex, the WLP methyl and carbonyl branch, the bifurcating hydrogenase, and the FDH gene cluster (Fig. 4D,E; Supplemental Fig. S3). These genes displayed twofold or greater transcriptional changes under A20, A10, and H10 growth conditions (average expression levels of 11852.7, 13491.7, and 12029.3, respectively) compared to the H20 growth conditions (average expression level, 3513.5) (Supplemental Table S5). Thus, this result suggests that the transcription of the acetogenesis genes in *A. bakii* is sensitive not only to the autotrophic growth conditions but also to low temperature. To verify whether the transcription of these genes is similarly affected by low temperatures in other acetogens, we independently compared the mRNA transcript abundance of the genes encoding *acsD*, *metF*, and *hydA* in *A. bakii* and *A. woodii* under autotrophic and low temperature conditions, using qRT-PCR (Fig. 4F). Whereas the *A. woodii* genes were significantly down-regulated under the H10 growth condition, the *A. bakii* genes were significantly up-regulated, suggesting that the response to cold stress may involve strain-specific transcriptional changes in the acetogenesis genes of psychrotolerant bacteria.

In contrast, the genes encoding enzymes of the glycolysis/gluconeogenesis pathway were significantly down-regulated or remained unchanged upon exposure to the H10 growth conditions, with fold-changes ranging from 0.01 to 0.68 (*P*_*adj*_ < 0.001) (Supplemental Table S5). Notably, three of the four genes encoding fructose-1,6-bisphosphate aldolase (FBA) were strongly down-regulated (from 0.01- to 0.11-fold, *P*_*adj*_ < 1.01 × 10^−63^) under the A20, H10, and A10 conditions. Interestingly, it was observed that the gene encoding glyceraldehyde-3-phosphate dehydrogenase (GAPDH) and one of the four genes encoding FBA were up-regulated under the H10 growth conditions with a fold change of 1.42 and 1.64, respectively (*P*_*adj*_ = 9.31 × 10^−6^ and *P*_*adj*_ = 2.39 × 10^−27^), similar to the autotrophic conditions. GAPDH displayed an important role in maintaining ATP pools under autotrophic conditions, according to previous transcriptome analysis performed in *C. autoethanogenum* (Marcellin et al. 2016). This result suggests that GAPDH potentially enhances cell growth at low temperature under autotrophic growth conditions. Taken together, our data showed that the low temperature represses the Embden–Meyerhof–Parnas (EMP) pathway, as is the case with *Lactococcus lactis* (Wouters et al. 2000) and *Bacillus subtilis* (Budde et al. 2006), and reinforced the WLP and the energy conservation system (Fig. 4E). Thus, the expression of genes involved in central carbon metabolism pathways, including the EMP and the WLP, is regulated by temperature, as well as carbon sources.

### Determination of the primary transcriptome landscape

**FIGURE 5.**
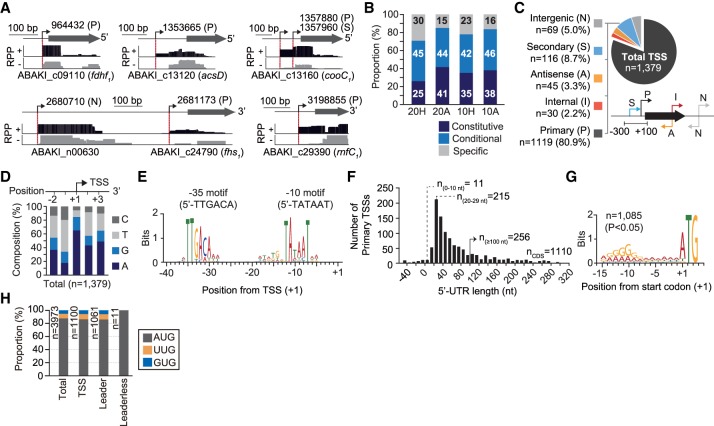
The transcription architecture of the *A. bakii* DSM 8239 genome. (*A*) Examples of dRNA-seq profiles mapped onto the *A. bakii* genome. Complementary DNA (cDNA) reads mapped to genome from RNA 5′-pyrophosphatase-treated (RPP+) and -untreated (RPP−) libraries are shown to represent the genomic regions. A red bar indicates the position of transcription initiation, which is identified by enriched 5′-ends of aligned dRNA-seq reads in RPP+ libraries (two biological duplicates) and nonenriched 5′-end reads of aligned dRNA-seq reads in RPP− libraries. See also Supplemental Figure S4A. (*B*) The proportion of constitutive (shared by all conditions), conditional (existing in two or more conditions), and specific (unique to a single condition) TSSs across the four conditions. (*C*) Categorization of 1379 TSSs identified in all samples. Primary TSSs (P) have a maximum peak density within a distance <300 bp upstream and 100 bp downstream from annotated genes; secondary TSSs (S) are associated with the same ORF as a nearby primary TSS but have lower peak density; internal TSSs (I) are located on the same strand as an annotation; antisense TSSs (A) are located on the opposite strand as an annotation; and intergenic TSSs (N) are those not falling into any of the above-mentioned categories. See Supplemental Table S6 for all TSS information and noncoding RNA lists, respectively. (*D*) Distribution of each nucleotide around the TSS (+1). (*E*) Motif searches upstream of *A. bakii* TSSs for all TSSs. See also Supplemental Figure S5. (*F*) Distribution of the 5′-UTR lengths of primary TSSs. (*G*) Conserved AG-rich Shine-Dalgarno (SD) sequences for 1085 protein-coding transcripts. (*H*) The frequency of start codons of all 3973 ORFs, 1100 TSS-assigned ORFs, 1061 ORFs containing 5′-UTRs (leader sequence), and 11 leaderless ORFs.

To further understand how gene expression is altered under autotrophic and cold conditions, we next analyzed the primary transcriptome of *A. bakii* by using the dRNA-seq method (Materials and Methods) (Singh and Wade 2014). Low-quality and adaptor sequences were trimmed and the trimmed sequence reads were mapped to the *A. bakii* genome, yielding at least 146× and 59× genome-scale coverage from RNA 5′-pyrophosphatase-treated (RPP+) and -untreated (RPP−) libraries, respectively (Supplemental Table S3). TSS positions were determined based on the ratio of 5′-end read intensities (greater than twofold) between the RPP+ and the RPP− libraries by using the in-house analytical workflow (Supplemental Fig. S4A; Rach et al. 2009; Jeong et al. 2016). For example, we identified the TSS positions of acetogenesis genes (Fig. 5A). A total of 951, 577, 681, and 637 TSSs were identified for the H20, A20, H10, and A10 conditions, respectively. To evaluate the reliability of the identified TSSs, independent verification was conducted by using the 5′ Rapid Amplification of cDNA Ends (5′RACE) and Sanger sequencing (Supplemental Fig. S4B,C).

Comparative analysis of the TSSs (1379 in total) yielded 239 constitutive TSSs (shared by all conditions), 514 conditional TSSs (shared by two or more conditions), and 626 specific TSSs (unique to a single condition) (Fig. 5B; Supplemental Table S6). Next, TSSs were further classified into five categories according to their genomic position and intensity (Fig. 5C). We detected 1119 primary TSSs (P) associated with 27.1% of the annotated genes and 116 secondary TSSs (S). In addition, by examining the putative operons constructed by the DOOR 2.0 operon database, we determined 1164 operons in the *A. bakii* genome, comprising 3155 ORFs (Supplemental Table S7; Mao et al. 2014). Among the computationally predicted operons, a total of 910 TSSs were mapped to 770 operons (∼66%). The incomplete TSS detection could be mainly due to the low cDNA concentration of the operons under the experimental conditions. A total of 13 TSSs were assigned to the acetogenesis clusters including genes of the WLP, the Rnf complex, the FDH complex, a bifurcating hydrogenase, and an ATP synthase (Supplemental Fig. S3). Interestingly, we observed one secondary TSS in a gene cluster for the carbonyl branch of the WLP, suggesting conditional initiation of transcription in response to specific environmental conditions. We further detected a total of 45 antisense TSSs (A) from the reverse strand of 42 genes, 30 internal TSSs (I) within 29 regions, and 69 intergenic TSSs (N) without any associated genes, confirming the presence of putative noncoding regulatory RNA molecules (Fig. 5C). Using the Cmsearch utility of INFERNAL (Nawrocki and Eddy 2013) and the Rfam database (Nawrocki et al. 2015), we found a total of 87 putative ncRNAs containing a TSS (Supplemental Table S8). Among these, 21 were primary TSSs, including two Moco RNA motifs and two FMN riboswitches located near the molybdopterin biosynthesis gene cluster (ABAKI_c09210 and ABAKI_c27150) and the riboflavin biosynthesis-associated genes (ABAKI_ c17190 and ABAKI_c21530), respectively. These ncRNAs are metabolite-dependent riboswitches that directly bind to the molybdenum cofactor or to FMN and are presumed to control the biosynthesis of molybdopterin and riboflavin by the 5′-UTR (Mironov et al. 2002; Regulski et al. 2008). A total of 19 TSSs were intergenic, including five cobalamin riboswitches, which are *cis*-regulatory elements modulating gene transcription or translation through the interaction with the 5′-UTR of the cobalamin (vitamin B12) biosynthesis/transport-related gene clusters (ABAKI_c14910 – c15410 and ABAKI_c39150) (Vitreschak et al. 2003).

Overall nucleotide preferences of the TSSs between the −2 to the +3 position were investigated (Fig. 5D). Preferences for a purine (A/G) at the position +1 and a pyrimidine (C/T) at the position −1 were found in diverse bacterial species (Cho et al. 2009; Sharma et al. 2010; Jeong et al. 2016). We found a similar dinucleotide preference as a purine (65.0% A and 19.6% G) and a pyrimidine (46.3% T and 19.8% C) were preferred for transcription initiation at the +1 and −1 TSS position, respectively. Therefore, our data were concordant with previous analyses at single-nucleotide resolution.

### Analysis of promoter and 5′-UTR sequences

The *A. bakii* genome encodes at least six sigma factors (RpoD, SigB, SigD, SigE, SigL, and SigH), which are key regulators orchestrating transcriptome changes in response to environmental conditions by the recognition of different core promoter sequences. To elucidate the principal promoter elements in the *A. bakii* genome, we analyzed the 50-bp sequences upstream of the identified TSSs, which were of sufficient length to cover the −10 and −35 boxes, using the MEME motif search algorithm (Bailey et al. 2009). As a result, 76.9%–88.5% and 30.4%–34.7% of the TSSs (*P* < 0.05; MEME) contained the consensus sequence of the extended −10 box motif (TATAAT) between −20 and −5 nt, and the −35 box motif (TTGACA) between −23 nt and −36 nt from the TSSs, respectively (Fig. 5E; Supplemental Fig. S5A–G). The spacer between the −10 and −35 motif was ∼17 bp. Taken together, our data suggest that most gene transcription is predominantly initiated by the RpoD housekeeping holoenzyme, including that of genes of the acetogenesis clusters (Supplemental Fig. S5H).

We next examined the 5′-UTR length distribution of mRNA transcripts. The median distance between the primary TSS to the start codon of protein-coding sequences (1110 in total) was 46 nt (Fig. 5F). More than 96% of the transcripts were found to have a 5′-UTR leader sequence longer than 10 nt and 23% of transcripts contained a leader sequence longer than 100 nt, potentially containing *cis*-regulatory elements (Bastet et al. 2011). The RBS motif and start codons were present in 1085 of 1110 5′-UTRs (97.7%) and were mainly represented by the “aggGGgg” sequence and ATG, respectively (Fig. 5G). The ATG codon was highly preferred as a translation start codon for both leader (957 genes) and leaderless (11 genes) mRNAs. The following most frequent start codons were TTG (89 genes) and GTG (64 genes), only utilized for leader transcripts (Fig. 5H).

### 5′-UTRs regulate the mRNA transcript levels of acetogenesis genes

**FIGURE 6.**
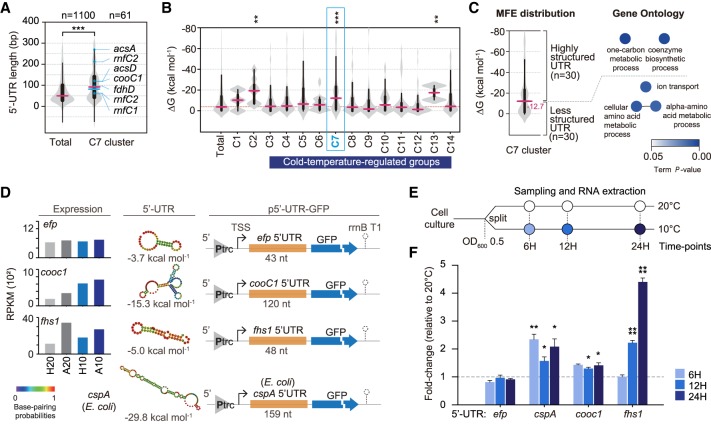
Low-temperature-responsive 5′-UTRs control the expression of acetogenesis-associated genes. (*A*) Comparison of the length distributions of 5′-UTRs between the total and C7 groups. The significance of differences was assessed by the Wilcoxon-Mann-Whitney test (***, *P* < 0.001). See also Supplemental Figure S6. (*B*) Comparison of the minimum folding free energy (MFE) distribution between the groups of DEG. Violin plots show median and quartile ranges of calculated MFEs. Secondary structures of a representative set of the mRNAs were predicted using the ViennaRNA RNAfold (Lorenz et al. 2011). The significance of differences was assessed by the Wilcoxon-Mann-Whitney test (**, *P* < 0.01; ***, *P* < 0.001). (*C*) Minimum folding free energy (MFE) distribution of C7 group and their Gene Ontology term enrichment analysis. Violin plots show median and quartile ranges of calculated MFE. Secondary structures of a representative set of the mRNAs were predicted using ViennaRNA RNAfold software. Enriched GO terms, including biological process, molecular function, and cellular component, were represented together as nodes. A Bonferroni-corrected *P* < 0.05 was considered the cut-off criterion. Term enrichment significance is represented by color. (*D*) Design of plasmid-based reporters for characterization of regulatory 5′-UTR regions. (*Left* panel) The transcriptional level of the *efp*, *cooc1*, and *fhs1* genes under the four growth conditions; (*middle* panel) secondary structure of each 5′-UTR. Structure and MFE were predicted using the ViennaRNA RNAfold software. (*Right* panel) Schematic illustration of the reporter gene constructs. Each segment of the gene 5′-UTRs of genes was fused to GFP under the constitutive *trc* promoter. Yellow boxes represent the specific mRNA 5′-UTRs. The sequence for the 5′-UTR of *cspA* was obtained from the genome of *E. coli* strain MG 1655 (NC_000913.3). (*E*) The assay design is shown. (*F*) Assays of 5′-UTR of *efp*, *cooc1*, *fhs1*, and *cspA* at 10°C and 20°C, respectively. The graph shows fold-changes in the GFP transcript abundance. Each value is the mean of three replicates in independently repeated experiments, and error bars show ±SEM. The *gyrA* gene was used as the reference (*) *P* < 0.05; (**) *P* < 0.01; (****) *P* < 0.0001 (Student's unpaired *t*-test).

5-UTRs can form a secondary structure influencing the expression of downstream genes, depending on metabolites, pH or temperature (Narberhaus et al. 2006). First, we examined the relationship between 5′-UTR lengths and gene expression patterns of all the groups of DEG (Supplemental Fig. S6). Interestingly, relatively long 5′-UTR lengths were observed for autotroph-specific groups, i.e., the clusters C1 (median length of 77 nt) and C2 (median length of 93 nt). The acetogenesis genes in cluster C7 showed a substantially wider range of 5′-UTR lengths (median length of 89 nt; *P* < 0.001; Wilcoxon-Mann-Whitney test) than other primary transcripts (Fig. 6A). To analyze the relationship between the secondary structure of 5′-UTR and the gene expression pattern, we computed the minimum free energy (MFE) and predicted the secondary structure of 5′-UTRs across the groups of DEGs using the ViennaRNA package 2.0 (Lorenz et al. 2011). The analysis showed that the clusters C1, C2, and C7 had markedly lower median ΔG values (−10.4, −19.5, and −12.3 kcal mol^−1^, respectively; *P* < 0.01, Wilcoxon-Mann-Whitney test) than those of the other clusters, whereas the cluster C12 showed a substantially higher median ΔG value (−1.4 kcal mol^−1^) (Fig. 6B). Based on these results, we anticipated that genes in the clusters C1, C2, and C7 are transcriptionally regulated by the secondary structures of their long 5′-UTR. Interestingly, the cluster C7 comprises two distinct gene populations with regard to the MFE of 5′-UTR. To understand the biological implications of these differences, we analyzed the functional profiles of the two gene populations using ClueGo (version 2.3.5) and obtained significantly distinct GO terms (Fig. 6C). In the case of the population displaying “highly structured” 5′-UTRs, two GO terms were significantly enriched, i.e., the “one-carbon metabolic process” and the “coenzyme biosynthetic process.” Different GO terms were enriched in the set of 30 genes with “less structured” 5′-UTR population, i.e., “ion transport,” “cellular amino acid metabolic process,” and “α-amino acid metabolic process.” To uncover *cis*-acting modulators of the two functionally distinct gene populations, 5′-UTRs were annotated using the Rfam database. Thirty-seven candidates were retrieved, which comprised 13 types of attenuation mechanisms including T-box, riboswitches, and ribosomal protein peptide leaders (Supplemental Table S9; Nudler and Mironov 2004; Choonee et al. 2007; Naville and Gautheret 2009).

Based upon the intrinsic termination mechanism of 5′-UTR *cis*-acting attenuators, we inferred the transcriptional state from the ratio between premature and full-length RNA transcript levels. When the *cis*-regulatory attenuator forms the closed state structure under low temperature conditions, transcription of the full-length mRNA may occur via the read-through mechanism (Supplemental Fig. S7A). We observed clearly distinct regulatory states for the tryptophan operon in the cluster C1 under heterotrophic and autotrophic growth conditions. Notably, tryptophan regulates the attenuator structure. Whereas, in the presence of adequate levels of tryptophan, the 5′-UTR and *trpE* displayed different transcript levels, when tryptophan level was low (e.g., autotrophic condition), we observed similar transcript levels (Supplemental Fig. S7B). Based on these findings, we examined whether the acetogenesis genes are regulated by transcriptional attenuation in response to temperature. However, such attenuation was not clearly observed (Supplemental Fig. S7C–G), suggesting that acetogenesis genes in the C7 cluster are not controlled by attenuators in response to temperature changes.

Long 5′-UTRs have been found to contribute to mRNA abundance and stability in bacteria (Arnold et al. 1998; Cao et al. 2014). For instance, the 5′-UTR of *cspA* in *Escherichia coli* has been extensively studied in terms of gene expression (Giuliodori et al. 2010). The *cspA* mRNA of *E. coli* is highly stable at low temperatures and its stability is substantially decreased at high temperatures (Goldenberg et al. 1996; Fang et al. 1997). Previous experiments revealed that the 5′-UTR of *cspA* mRNA plays a critical role in the temperature-dependent changes in transcript stability (Brandi et al. 1996), whereas the CspA promoter is not required for cold induction (Fang et al. 1997). To confirm the relationship between the 5′-UTR secondary structure and cold induction of acetogenesis gene expression, we designed a plasmid-based reporter system containing a *gfp* gene controlled by a constitutive promoter and by the 5′-UTR of the selected genes (p5′-UTR-GFP; Fig. 6D). To this end, the 5′-UTRs of *cooC1* (120 nt) and *fhs1* (48 nt), derived from the leading genes of the WLP methyl and carbonyl branches, respectively, were used. Plasmid-based reporters containing the 43-nt long 5′-UTR of *efp* (elongation factor P) and the 159-nt long 5′-UTR of *cspA* of *E. coli* (cold shock protein A) were also constructed and utilized as negative and positive controls, respectively (Fig. 6D). Because the detailed investigation of the metabolic or regulatory networks of *A. bakii* is currently limited by the absence of available genetic tools, we used an *E. coli* DH5α strain harboring the above-mentioned 5′-UTR-GFP plasmid. The amount of mRNA transcripts of the *gfp* gene from the four plasmids was then quantified at 20°C and 10°C using qRT-PCR (Fig. 6E). As expected, the 5′-UTR of *cspA* displayed temperature-dependent regulation, showing a significantly higher mRNA level (fold change >1.6, *P* < 0.05) at 10°C compared to 20°C (Fig. 6F). Intriguingly, the assay showed statistically significant changes in the mRNA levels of *cooc1* (fold change >1.3, *P* < 0.05) and *fhs1* (fold change >2.2, *P* < 0.0001) at the 12 and 24 h time points, indicating that the 5′-UTR structure positively affected mRNAs stability at 10°C (Fig. 6F). On the other hand, no significant changes in *efp* mRNA abundance were observed (*P* > 0.05) at any time point. Taken together, our data showed that the mRNA levels of *cooc1* and *fhs1* were significantly up-regulated at low temperature and that the long 5′-UTRs were involved in the thermoregulation of acetogenesis gene expression. The results imply that the RNA structure may mediate the response to environmental factors, including temperature, by the activation of multiple physiological processes, playing an important role in the cold-dependent post-transcriptional regulation under autotrophic conditions.

## DISCUSSION

In this study, we completed the genome sequence of the psychrotolerant acetogen *Acetobacterium bakii* DSM 8239 (4.28 Mbp in size) and revealed transcriptomes specifically associated to autotrophic growth at low temperature. Moreover, we identified key regulators underlying the transcriptional response to these environmental conditions, as well as the complete WLP of *A. bakii*. Determination of the *A. bakii* primary transcriptome provided insights into transcriptional regulation under physiological conditions. Comparative genomic analysis revealed that, although acetogenesis is fundamentally similar in *A. bakii* and *A. woodii* (Poehlein et al. 2012; Schuchmann and Müller 2013, 2014), some differences exist, such as a truncated selenium-free FDH subunit and a distinct configuration of the subunit c-ring of F_1_F_0_ ATP synthase (Matthies et al. 2014).

Differential gene expression data obtained under cold and autotrophic growth conditions showed that the acetogenesis genes are activated in response not only to the autotrophic conditions but also to low temperature. This unique transcriptional regulation is not present in the phylogenetically related mesophilic strain *A. woodii*. Interestingly, a group of 252 genes, including acetogenesis genes, contained longer 5′-UTRs (with a median length of 89 nt) with lower RNA folding free energy as compared to the other genes (with a median 5′-UTR length of 46 nt). Based on these findings, we propose that the transcription of acetogenesis genes is activated by cold temperature via RNA conformational changes in the long 5′-UTR, similar to the cold shock induction of the *cspA* gene in *E. coli*. Because the 5′ stem-loop of the *cspA* mRNA is only moderately stable above 30°C (Hankins et al. 2007), the *cspA* mRNA is rapidly degraded at higher temperatures. However, cold temperature stabilizes the 5′-UTR of the *cspA* mRNA by blocking its interaction with the sensor domain of RNase E (Emory et al. 1992; Goldenberg et al. 1996; Fang et al. 1997; Callaghan et al. 2005). Therefore, the cold-dependent induction of acetogenesis genes may result from a coordinated activity of 5′–3′ exo- or endo-ribonuclease activity (e.g., RNase E, RNase Y, and RNase J), which showed mRNA structure-dependent endonuclease activity (Goldenberg et al. 1996; Hui et al. 2014). These ribonucleases are highly expressed under all tested conditions (Supplemental Table S4), and may act in concert on 5′-leaders to regulate the mRNA stability of acetogenesis genes. These results indicate that the 5′-UTRs may represent regulatory elements functioning as bacterial RNA thermometers (Kortmann and Narberhaus 2012) responding to temperature variations in psychrophilic and psychrotolerant microbes. Recent studies in the distantly related member of methanogenic archaea, *Methanolobus psychrophilus*, also showed that 5′-UTR-mediated transcriptional and post-transcriptional regulations play a critical role in the bacterial response to cold (Li et al. 2015; Qi et al. 2017).

In conclusion, this study suggests that psychrotolerant acetogens can activate acetogenesis metabolism even under cold heterotrophic conditions. The transcriptome changes of the psychrotolerant acetogens, in response to CO_2_/H_2_ and low temperature, suggest the importance of post-transcriptional regulation in cold-active acetogenesis. This is a robust and low-energy-cost mechanism for sensing temperature variations and regulating the mRNA levels accordingly (Naville and Gautheret 2009). This energetically favorable regulation of gene expression is expected to be crucial for psychrophilic and psychrotolerant bacteria living in cold habitats, which are extremely low-energy environments (Hoehler and Jørgensen 2013). However, the molecular details of this mechanism are not yet well understood and further studies focusing on the described RNA-based events will broaden our comprehension of cold-induced gene regulation and of the role played by the genes of the acetogenesis metabolic pathway.

## MATERIALS AND METHODS

### Bacterial strain and growth conditions

*Acetobacterium bakii* DSM 8239 was obtained from the Leibniz Institute DSMZ-German Collection of Microorganisms and Cell Cultures (DSMZ). *A. bakii* cells were cultured strictly anaerobically at two temperatures, 10°C and 20°C, representing cold stress and optimal conditions, respectively, in DSMZ Medium 135 (Atlas 2010). *A. woodii* cells were cultured at two temperatures, 20°C and 30°C, representing cold and optimal conditions, respectively. DSM medium 135 without fructose and resazurin was prepared and supplemented with 2 g/L NaCl. For growth, a headspace pressure of 200 kPa under a gas mixture of H_2_/CO_2_ (80/20, v/v) provided autotrophic conditions, and 5 g/L fructose provided heterotrophic conditions.

### Analytical methods

Cell growth was monitored by measuring the optical density (OD_600_) and metabolic products were analyzed by high-performance liquid chromatography (HPLC). The concentrations of acetate and other organic acids were routinely determined using a Waters HPLC 1525 system (Waters) equipped with a refractive index detector (RID, Waters) operated at 37°C and a MetaCarb 87H organic acid column (Agilent Technologies). Slightly acidified water (0.01 N H_2_SO_4_) was used as the mobile phase, with a flow rate of 0.6 mL/min. To remove proteins and other cell residues, 500 µL samples were centrifuged at 14,000*g* for 10 min at 4°C and all samples were filtered through a 0.2-µm Minisart RC15 Syringe Filters (Sartorius). The supernatant (20 µL) was then injected into the HPLC system for analysis.

### Genomic DNA extraction and sequencing

A bacteria culture was centrifuged at 3000*g* at 4°C for 15 min and ground using a mortar and a pestle kept in liquid nitrogen. Total genomic DNA extraction was performed using the Genomic-tip 500/G kit (Qiagen) and the Genomic DNA Buffer Set (Qiagen). DNA quality and integrity were evaluated by the A260/A280 ratio (>1.9) and agarose gel electrophoresis of genomic DNA. DNA quantity was measured by a spectrophotometer using the NanoDrop 2000 platform (Thermo Scientific). A library was constructed using the PacBio (Pacific Biosciences) 20-kb library preparation protocol and sequencing was performed using a PacBio RS II with P6-C4 chemistry. Low-quality reads (min. read quality <0.8) were filtered out and de novo assembly was conducted using the hierarchical genome assembly process (HGAP, v2.3) workflow (Chin et al. 2013), including consensus polishing with Quiver. As a result of the HGAP process, we obtained a total length of 4,316,241 bp and five contigs after the polishing process. For genome assembly, scaffolding tasks were performed using SSPACE (Hunt et al. 2014) with mate-pair sequences and the PCR-based primer walking method. Highly accurate short reads generated from our previous study (Hwang et al. 2015), and ssRNA-seq data, were used to improve the accuracy of PacBio scaffolds. Short-read polishing was performed by CLC Genomics Workbench v6.5.1 with the following alignment parameters: mismatch cost = 2, deletion cost = 3, insertion cost = 3, length fraction = 0.9, and similarity fraction = 0.9. The location of conflicting sequences was then detected by the Probabilistic Variant Detection toolbox of the CLC Genomics Workbench, using the default parameters.

### Gene prediction and annotation

Briefly, the coding sequences (CDSs) were predicted using Prodigal v.2.6.3 (Hyatt et al. 2010). To assign protein functions, predicted CDSs were annotated with the best BLASTP hits from integrated databases with UniProt/SwissProt/KEGG/GO and UniProt/TrEMBL databases (UniProt Consortium 2007; Gene Ontology Consortium 2015; Kanehisa et al. 2016). RNAmmer v1.2 (Lagesen et al. 2007), tRNAscan SE v2.0 (Lowe and Chan 2016), and Infernal v1.1.2 (Nawrocki and Eddy 2013) with Rfam database v12.1 (Nawrocki et al. 2015) were used to identify rRNAs, tRNAs, and noncoding RNAs, respectively. The CRISPR array was identified by CRT v1.1 (Bland et al. 2007). The predicted proteins from the annotation pipeline were evaluated with HMMScan to identify Pfam domains using the default gathering thresholds from the Pfam database v30.0 (Finn et al. 2016).

### Resting cell assay

All the steps were performed under strictly anoxic conditions (O_2_ ppm < 5) in an anaerobic chamber (Coy Laboratory product) filled with 96% N_2_ and 4% H_2_. Cells were cultured under strict anaerobic conditions in 10× 100 mL DSMZ Medium 135 with 5 g/L fructose to an OD_600_ of 0.4. A bacteria culture was centrifuged at 12,000*g* at 4°C for 15 min and washed twice with imidazole buffer (50 mM imidazole-HCl, 20 mM MgSO_4_, 20 mM KCl, 4.4 µM resazurin, 4 mM DTE, pH 7.0). The cells were resuspended to a protein concentration of 25 mg/mL and transferred to Hungate tubes, and the gas phase of the cell suspensions was changed to N_2_. For the resting cell assay, the cells were resuspended in the same imidazole buffer to a protein concentration of 0.25 mg/mL in serum bottles. NaCl (30 mM) was added as indicated. Subsequently, the gas phase of the cell suspensions was changed to H_2_-CO_2_ (80:20, v/v, 200 kPa) or N_2_ (200 kPa). The cell suspensions were incubated at 20°C without shaking. 900 µL samples were taken at indicated time points and cells were removed by centrifugation immediately at 12,000*g* at 4°C for 10 min. Acetate concentrations were determined using a Waters HPLC 1525 system (Waters).

### RNA-extraction and RNA-sequencing (RNA-seq)

For RNA extraction, cultures of heterotrophically and autotrophically grown cells were harvested at OD_600_ of 0.6 and 0.08, respectively. Next, 400 and 800 mL of the cultures were immediately harvested by centrifugation at 3000*g* at 4°C for 15 min, and resuspended in 500 µL of cell lysis buffer (140 mM NaCl, 20 mM Tris-HCl pH 7.4, 5 mM MgCl_2_, and 1% Triton X-100). Resuspended cells were flash frozen and ground using a mortar and pestle containing liquid nitrogen for all experiments including RNA-seq, dRNA-seq, and qRT-PCR. The cell debris and unbroken cells were removed by centrifugation at 4000*g* for 10 min at 4°C. Total RNA was then isolated using the TRIzol reagent (Thermo Scientific) according to the manufacturer's instructions. To obtain DNA-free RNA, all RNA samples were treated with rDNase I (Ambion), according to the manufacturer's instructions. The quantity of total RNA was measured with a NanoDrop 2000 spectrophotometer (Thermo Scientific), the quality of total RNA was evaluated by the A260/A280 ratio (>1.8) and the integrity of the ribosomal RNAs (rRNA) bands was assessed by 2% agarose gel electrophoresis. Ribosomal RNAs were specifically removed by Ribo-Zero rRNA Removal Kit bacteria (Illumina) following the manufacturer's instructions, and the rRNA quality was evaluated by agarose gel electrophoresis. RNA-seq libraries were prepared using Illumina's TruSeq Strand mRNA LT Sample Prep Kit (Illumina) according to the manufacturer's instructions and then sequenced using an Illumina Hi-Seq 2500 instrument (Rapid-Run mode) with a 50-bp single-end sequencing recipe.

### Differential RNA-seq (dRNA-seq) library preparation

Four hundred nanograms of rRNA-depleted RNA were split into two samples for the preparation of two different libraries (RPP+ and RPP−). The RNA concentration was measured by a Qubit RNA HS Assay Kit. Two dRNA-seq libraries were prepared using a modified protocol as described previously (Singh and Wade 2014). Briefly, the 200 ng intended for the RPP+ library, containing both primary (5′-PPP) and processed (5′-P/5′-OH) transcripts, were treated with RNA 5′-polyphosphatase (Epicentre). In the 200 ng intended for the RPP- library, enriched in processed transcripts, RNA 5′-polyphosphatase was not used. 5′-RNA adaptors were ligated to RNA using T4 RNA ligase (Thermo) and purified with Agencourt AMPure XP beads (Beckman Coulter). 5′-RNA adaptors were treated with alkaline phosphatase (Thermo) before ligation, with a 1:3 RNA to 5′-RNA adaptor molar ratio for ligation. Reverse transcription was performed using random nonamer adaptors (Supplemental Table S10) and SuperScript III Reverse Transcriptase kit (Invitrogen) following the manufacturer's directions. Thirty microliters of nuclease-free water were added to the synthesized cDNA sample, and the cDNA libraries were purified with a 0.8× volume of AMPure XP beads. PCR amplification of the sequencing library was performed with a Fusion High-Fidelity polymerase (Thermo). The PCR conditions were as follows: 98°C for 30 sec; several cycles (monitored real-time) of 98°C for 10 sec, 56°C for 20 sec, and 72 °C for 20 sec; and followed by 72°C for 5 min. The amplification was monitored with SYBR green gel stain solution (Invitrogen) on a CFX96 Real-Time PCR Detection System (Bio-Rad) until the PCR reaction reached the plateau phase. The amplified dRNA-seq libraries were purified with a 0.8× volume of AMPure XP beads. The library concentration was measured by the Qubit DNA HS assay kit, and dRNA-seq libraries were sequenced using the 100-bp read recipe by an Illumina HiSeq2500 (Illumina). Two independent biological experiments were performed. All oligo sequences are available in Supplemental Table S10.

### 5′ rapid amplification of cDNA ends (5′ RACE) analysis

The selected TSSs, the expected amplicon sizes, and the sequences of primary mRNAs are shown in Supplemental Figure S5C. 5′ RACE analysis was conducted according to previously published protocols (Jeong et al. 2017). The target-specific RACE primers for 5′-end mapping are available in Supplemental Table S10. Amplified products were analyzed on a 2% agarose gel and the results were further confirmed using conventional Sanger sequencing.

### RNA-seq data analysis

The sequence reads obtained from RNA-seq (50-bp single-end reads) and dRNA-seq (100-bp single-end reads) were analyzed using the CLC Genomics Workbench 6.5.1 (Qiagen). Bases with low quality and adaptor sequences were trimmed and aligned to the *A. bakii* genome with the following parameters: mismatch cost = 2, insertion cost = 3, deletion cost = 3, length fraction = 0.9, and similarity fraction = 0.9. For RNA-seq data, a raw read count table was constructed from RNA-seq reads using CLC's RNA-seq analysis module. To perform the normalization and differential gene expression analysis, the count table was used as input file for the Bioconductor package DEseq2 (Love et al. 2014). To identify the differently expressed genes, the significant levels were set at a false discovery rate (FDR) < 0.01 and log_2_-fold change (log_2_ FC) > |1|.

### dRNA-seq data analysis

For dRNA-seq data, the analytical workflow is graphically presented in Supplemental Figure S5A. We applied a scaling normalization factor to the 5′-ends of the aligned dRNA-seq read profiles. Since the RPP+ (RNA 5′-polyphosphatase-treated) library also contains primary transcripts, both libraries should appear to have equal gene expression within genes. For this approach, the scaling factor of each library was calculated by the matrix of the read count produced by the RNA-seq analysis module in CLC and the estimateSizeFactors function implemented in the DEseq2 package. Genomic coordinates for the 5′-ends of aligned dRNA-seq reads were considered to be potential transcription start sites (TSSs). TSSs were identified and curated by comparison between the RPP+ and the RPP− libraries as described previously with some modifications (Rach et al. 2009; Jeong et al. 2016). Briefly, initial peaks were clustered within 300 bp; they were then sub-clustered based on a standard deviation <15. If an initial cluster had multiple peaks, only the standard deviation of two adjacent peaks was calculated. The potential TSS with the highest peak density was used as the TSS in sub-cluster peaks. The TSSs were then selected based on the ratio of peak densities in the RPP+ and TAP− (5′-polyphosphatase-untreated) libraries (greater than twofold difference) and on biological reproducibility. The selected TSSs were manually curated and classified into five categories based on their genomic position. Primary TSSs (P) were defined as TSSs with a maximum peak density within a distance <300 bp upstream and <100 bp downstream from annotated genes; secondary TSSs (S) were associated with the same ORFs as nearby primary TSSs but had a lower peak density; internal TSSs (I) were those located on the same strand as an annotation; antisense TSSs (A) were located on the opposite strand as an annotation; and intergenic TSSs (N) were those not included in any of the above-mentioned categories. The TSSs were compared among the four conditional libraries, and summed as total TSSs within the range of ±4 bp. All TSSs associated with annotated genes are indicated in Supplemental Table S6. In-house Perl scripts used for TSS determination and classification are available at http://cholab.or.kr/data/. Multilayered RNA-seq and dRNA-seq data were visualized using the NimbleGen's SignalMap software.

### Quantitative RT-PCR assay

Primers were designed using the NCBI Primer-Blast software and are available in Supplemental Table S10. The purified RNA samples from *A. bakii* or *A. woodii* were reversely transcribed to cDNA using the SuperScript III First-Strand Synthesis System (Invitrogen), following the manufacturer's directions. Relative expression of the mRNAs was determined using a KAPA SYBR FAST Universal qPCR kit (Kapa Biosystems) following the manufacturer's instructions.

### Operon prediction

To define an operon map of *A. bakii* genome, we integrated putative operons generated by the DOOR 2.0 operon database (Mao et al. 2014) with dRNA-seq and RNA-seq data. A total of 770 putative operons were manually validated by examining the presence of TSSs at the upstream region of each operon and their cDNA coverage. The remaining 394 operons were refined by RNA-seq data. The operons are summarized in Supplemental Table S7.

### Motif detection in promoters

The 50 nt of DNA sequences upstream of each determined TSS were extracted. The conserved sequence motifs of the −10 and −35 elements were analyzed by MEME v4.11.1 (Bailey et al. 2009) (MEME *P* < 0.05 was considered as the cut-off criterion).

### Construction of p5′-UTR-GFP reporter plasmids

Oligonucleotides and DNA fragments used in this study are listed in Supplemental Table S10. To construct pGFP reporter plasmids, *gfp*+*rrnB* T1 fragment was amplified by PCR from pHCE-eGFP plasmid using primers pHCE_EGFP-rrnBT_F/pHCE_EGFP-rrnBT_R. PCR product was gel-purified and cloned into the *Nde*I and *EcoR*I sites of pUC19 using the In-Fusion HD cloning kit (Clontech). To generate four p5′-UTR-GFP reporter plasmids, four trc promoter + 5′-UTR modules were synthesized by Integrated DNA Technologies (IDT), and each trc promoter + 5′-UTR module was cloned into the *BamH*I and *Nco*I sites of the pGFP plasmid using the In-Fusion HD cloning kit (Clontech). The constructs were used to transform competent *E. coli* DH5α and ampicillin-resistant transformants were selected.

### Assays for p5′-UTR-GFP reporter constructs

*E. coli* DH5α cells carrying p5′-UTR-GFP reporter plasmids were inoculated in 50 mL of LB with ampicillin at 30°C. When cells reached OD_600_ of 0.5, cultures were split into two samples, and incubated at 10°C and 20°C, respectively. Three milliliters of the cultures were withdrawn at three time points (6, 12, and 24 h), immediately harvested by centrifugation at 3000*g* at 4°C for 15 min and each RNA was immediately extracted. The amount of GFP transcript associated with the 5′-UTR of each gene was measured by qPCR. The GyrA gene of *E. coli* was used as a reference gene in qRT-PCR.

### Statistical testing

All statistical testing (Student's *t*-test, Wilcoxon-Mann-Whitney test, and Wilcoxon matched-pairs signed rank test) was done using the GraphPad Prism v8 software. *P*-values <0.05 were considered as statistically significant.

## DATA DEPOSITION

The genome was submitted to the European Nucleotide Archive (ENA) with sample ID UHJK01000000. This ENA annotation information is automatically generated through the ENA Genome Annotation Pipeline and may differ from the annotation used for this manuscript. The genome annotations used for this manuscript are available at http://cholab.or.kr/data/. Total transcriptome data in FASTQ format are available in the ENA database with the study accession number PRJEB21929.

## SUPPLEMENTAL MATERIAL

Supplemental material is available for this article.

## Supplementary Material

Supplemental Material

## References

[RNA068239SHIC1] Aguilar PS, Hernandez-Arriaga AM, Cybulski LE, Erazo AC, de Mendoza D. 2001 Molecular basis of thermosensing: a two-component signal transduction thermometer in *Bacillus subtilis*. EMBO J 20: 1681–1691. 11285232 10.1093/emboj/20.7.1681 PMC145467

[RNA068239SHIC2] Arnold TE, Yu J, Belasco JG. 1998 mRNA stabilization by the ompA 5′ untranslated region: two protective elements hinder distinct pathways for mRNA degradation. RNA 4: 319–330. 9510333 PMC1369620

[RNA068239SHIC3] Atlas RM. 2010 Composition of media. In Handbook of microbiological media, 4th ed., pp. 11–170. CRC Press, Boca Raton, FL.

[RNA068239SHIC4] Bailey TL, Boden M, Buske FA, Frith M, Grant CE, Clementi L, Ren J, Li WW, Noble WS. 2009 MEME SUITE: tools for motif discovery and searching. Nucleic Acids Res 37: W202–W208. 19458158 10.1093/nar/gkp335 PMC2703892

[RNA068239SHIC5] Barria C, Malecki M, Arraiano CM. 2013 Bacterial adaptation to cold. Microbiology 159: 2437–2443. 24068238 10.1099/mic.0.052209-0

[RNA068239SHIC6] Bastet L, Dubé A, Massé E, Lafontaine DA. 2011 New insights into riboswitch regulation mechanisms. Mol Microbiol 80: 1148–1154. 21477128 10.1111/j.1365-2958.2011.07654.x

[RNA068239SHIC7] Bengelsdorf FR, Straub M, Dürre P. 2013 Bacterial synthesis gas (syngas) fermentation. Environ Technol 34: 1639–1651. 24350425 10.1080/09593330.2013.827747

[RNA068239SHIC8] Bland C, Ramsey TL, Sabree F, Lowe M, Brown K, Kyrpides NC, Hugenholtz P. 2007 CRISPR recognition tool (CRT): a tool for automatic detection of clustered regularly interspaced palindromic repeats. BMC Bioinformatics 8: 209. 17577412 10.1186/1471-2105-8-209 PMC1924867

[RNA068239SHIC9] Bowman JP. 2008 Genomic analysis of psychrophilic prokaryotes. In Psychrophiles: from biodiversity to biotechnology (ed. Margasin R, ), pp. 265–284. Springer-Verlag, Berlin/Heidelberg, Germany.

[RNA068239SHIC10] Brandi A, Pietroni P, Gualerzi CO, Pon CL. 1996 Post-transcriptional regulation of CspA expression in *Escherichia coli*. Mol Microbiol 19: 231–240. 8825769 10.1046/j.1365-2958.1996.362897.x

[RNA068239SHIC11] Brown SD, Nagaraju S, Utturkar S, De Tissera S, Segovia S, Mitchell W, Land ML, Dassanayake A, Köpke M. 2014 Comparison of single-molecule sequencing and hybrid approaches for finishing the genome of *Clostridium autoethanogenum* and analysis of CRISPR systems in industrial relevant Clostridia. Biotechnol Biofuels 7: 40. 24655715 10.1186/1754-6834-7-40 PMC4022347

[RNA068239SHIC12] Budde I, Steil L, Scharf C, Völker U, Bremer E. 2006 Adaptation of *Bacillus subtilis* to growth at low temperature: a combined transcriptomic and proteomic appraisal. Microbiology 152: 831–853. 16514163 10.1099/mic.0.28530-0

[RNA068239SHIC13] Callaghan AJ, Marcaida MJ, Stead JA, McDowall KJ, Scott WG, Luisi BF. 2005 Structure of *Escherichia coli* RNase E catalytic domain and implications for RNA turnover. Nature 437: 1187–1191. 16237448 10.1038/nature04084

[RNA068239SHIC14] Cao Y, Li J, Jiang N, Dong X. 2014 Mechanism for stabilizing mRNAs involved in methanol-dependent methanogenesis of cold-adaptive *Methanosarcina mazei* zm-15. Appl Environ Microbiol 80: 1291–1298. 24317083 10.1128/AEM.03495-13 PMC3911069

[RNA068239SHIC15] Chin C-S, Alexander DH, Marks P, Klammer AA, Drake J, Heiner C, Clum A, Copeland A, Huddleston J, Eichler EE, 2013 Nonhybrid, finished microbial genome assemblies from long-read SMRT sequencing data. Nat Methods 10: 563–569. 23644548 10.1038/nmeth.2474

[RNA068239SHIC16] Cho B-K, Zengler K, Qiu Y, Park YS, Knight EM, Barrett CL, Gao Y, Palsson BØ. 2009 The transcription unit architecture of the *Escherichia coli* genome. Nat Biotechnol 27: 1043–1049. 19881496 10.1038/nbt.1582 PMC3832199

[RNA068239SHIC17] Choonee N, Even S, Zig L, Putzer H. 2007 Ribosomal protein L20 controls expression of the *Bacillus subtilis* infC operon via a transcription attenuation mechanism. Nucleic Acids Res 35: 1578–1588. 17289755 10.1093/nar/gkm011 PMC1865079

[RNA068239SHIC18] Darling AE, Mau B, Perna NT. 2010 progressiveMauve: multiple genome alignment with gene gain, loss and rearrangement. PLoS One 5: e11147. 20593022 10.1371/journal.pone.0011147 PMC2892488

[RNA068239SHIC19] Drake HL. 1995 Acetogenesis, acetogenic bacteria, and the acetyl-CoA “Wood/Ljungdahl” pathway: past and current perspectives. In Acetogenesis, pp. 3–60. Springer US, New York.

[RNA068239SHIC20] Drake HL, Küsel K, Matthies C. 2006 Acetogenic prokaryotes. In The prokaryotes, pp. 354–420. Springer, New York.

[RNA068239SHIC21] Emory SA, Bouvet P, Belasco JG. 1992 A 5′-terminal stem-loop structure can stabilize mRNA in *Escherichia coli*. Genes Dev 6: 135–148. 1370426 10.1101/gad.6.1.135

[RNA068239SHIC22] Fang L, Jiang W, Bae W, Inouye M. 1997 Promoter-independent cold-shock induction of *cspA* and its derepression at 37°C by mRNA stabilization. Mol Microbiol 23: 355–364. 9044269 10.1046/j.1365-2958.1997.2351592.x

[RNA068239SHIC23] Finn RD, Coggill P, Eberhardt RY, Eddy SR, Mistry J, Mitchell AL, Potter SC, Punta M, Qureshi M, Sangrador-Vegas A, 2016 The Pfam protein families database: towards a more sustainable future. Nucleic Acids Res 44: D279–D285. 26673716 10.1093/nar/gkv1344 PMC4702930

[RNA068239SHIC24] Gene Ontology Consortium. 2015 Gene Ontology Consortium: going forward. Nucleic Acids Res 43: D1049–D1056. 25428369 10.1093/nar/gku1179 PMC4383973

[RNA068239SHIC25] Giuliodori AM, Di Pietro F, Marzi S, Masquida B, Wagner R, Romby P, Gualerzi CO, Pon CL. 2010 The cspA mRNA is a thermosensor that modulates translation of the cold-shock protein CspA. Mol Cell 37: 21–33. 20129052 10.1016/j.molcel.2009.11.033

[RNA068239SHIC26] Goldenberg D, Azar I, Oppenheim AB. 1996 Differential mRNA stability of the cspA gene in the cold-shock response of *Escherichia coli*. Mol Microbiol 19: 241–248. 8825770 10.1046/j.1365-2958.1996.363898.x

[RNA068239SHIC27] Hankins JS, Zappavigna C, Prud'homme-Généreux A, Mackie GA. 2007 Role of RNA structure and susceptibility to RNase E in regulation of a cold shock mRNA, cspA mRNA. J Bacteriol 189: 4353–4358. 17416651 10.1128/JB.00193-07 PMC1913359

[RNA068239SHIC28] Henstra AM, Sipma J, Rinzema A, Stams AJ. 2007 Microbiology of synthesis gas fermentation for biofuel production. Curr Opin Biotechnol 18: 200–206. 17399976 10.1016/j.copbio.2007.03.008

[RNA068239SHIC29] Hoehler TM, Jørgensen BB. 2013 Microbial life under extreme energy limitation. Nat Rev Microbiol 11: 83–94. 23321532 10.1038/nrmicro2939

[RNA068239SHIC30] Hui MP, Foley PL, Belasco JG. 2014 Messenger RNA degradation in bacterial cells. Annu Rev Genet 48: 537–559. 25292357 10.1146/annurev-genet-120213-092340 PMC4431577

[RNA068239SHIC31] Hunt M, Newbold C, Berriman M, Otto TD. 2014 A comprehensive evaluation of assembly scaffolding tools. Genome Biol 15: R42. 24581555 10.1186/gb-2014-15-3-r42 PMC4053845

[RNA068239SHIC32] Hwang S, Song Y, Cho B-K. 2015 Draft genome sequence of *Acetobacterium bakii* DSM 8239, a potential psychrophilic chemical producer through syngas fermentation. Genome Announc 3: e01070-15. 26404601 10.1128/genomeA.01070-15 PMC4582577

[RNA068239SHIC33] Hyatt D, Chen G-L, LoCascio PF, Land ML, Larimer FW, Hauser LJ. 2010 Prodigal: prokaryotic gene recognition and translation initiation site identification. BMC Bioinformatics 11: 119. 20211023 10.1186/1471-2105-11-119 PMC2848648

[RNA068239SHIC34] Jeong Y, Kim J-N, Kim MW, Bucca G, Cho S, Yoon YJ, Kim B-G, Roe J-H, Kim SC, Smith CP, 2016 The dynamic transcriptional and translational landscape of the model antibiotic producer *Streptomyces coelicolor* A3(2). Nat Commun 7: 11605. 27251447 10.1038/ncomms11605 PMC4895711

[RNA068239SHIC35] Jeong Y, Shin H, Seo SW, Kim D, Cho S, Cho B-K. 2017 Elucidation of bacterial translation regulatory networks. Curr Opin Syst Biol 2: 84–90.

[RNA068239SHIC36] Kanehisa M, Sato Y, Kawashima M, Furumichi M, Tanabe M. 2016 KEGG as a reference resource for gene and protein annotation. Nucleic Acids Res 44: D457–D462. 26476454 10.1093/nar/gkv1070 PMC4702792

[RNA068239SHIC37] Köpke M, Held C, Hujer S, Liesegang H, Wiezer A, Wollherr A, Ehrenreich A, Liebl W, Gottschalk G, Dürre P. 2010 *Clostridium ljungdahlii* represents a microbial production platform based on syngas. Proc Natl Acad Sci 107: 13087–13092. 20616070 10.1073/pnas.1004716107 PMC2919952

[RNA068239SHIC38] Kortmann J, Narberhaus F. 2012 Bacterial RNA thermometers: molecular zippers and switches. Nat Rev Microbiol 10: 255–265. 22421878 10.1038/nrmicro2730

[RNA068239SHIC39] Kotsyurbenko OR, Simankova MV, Available ANNN, Zhilina TN, Bolotina NP, Lysenko AM, Osipov GA. 1995 New species of psychrophilic acetogens: *Acetobacterium bakii* sp. nov., *A. paludosum* sp. nov., *A. fimetarium* sp. nov. Arch Microbiol 163: 29–34.

[RNA068239SHIC40] Kotsyurbenko OR, Glagolev MV, Nozhevnikova AN, Conrad R. 2001 Competition between homoacetogenic bacteria and methanogenic archaea for hydrogen at low temperature. FEMS Microbiol Ecol 38: 153–159.

[RNA068239SHIC41] Kurtz S, Phillippy A, Delcher AL, Smoot M, Shumway M, Antonescu C, Salzberg SL. 2004 Versatile and open software for comparing large genomes. Genome Biol 5: R12. 14759262 10.1186/gb-2004-5-2-r12 PMC395750

[RNA068239SHIC42] Lagesen K, Hallin P, Rødland EA, Staerfeldt HH, Rognes T, Ussery DW. 2007 RNAmmer: consistent and rapid annotation of ribosomal RNA genes. Nucleic Acids Res 35: 3100–3108. 17452365 10.1093/nar/gkm160 PMC1888812

[RNA068239SHIC43] Latif H, Zeidan AA, Nielsen AT, Zengler K. 2014 Trash to treasure: production of biofuels and commodity chemicals via syngas fermenting microorganisms. Curr Opin Biotechnol 27: 79–87. 24863900 10.1016/j.copbio.2013.12.001

[RNA068239SHIC44] Li J, Qi L, Guo Y, Yue L, Li Y, Ge W, Wu J, Shi W, Dong X. 2015 Global mapping transcriptional start sites revealed both transcriptional and post-transcriptional regulation of cold adaptation in the methanogenic archaeon *Methanolobus psychrophilus*. Sci Rep 5: 9209. 25784521 10.1038/srep09209 PMC5378194

[RNA068239SHIC45] Lorenz R, Bernhart SH, Höner Zu Siederdissen C, Tafer H, Flamm C, Stadler PF, Hofacker IL. 2011 ViennaRNA Package 2.0. Algorithms Mol Biol 6: 26. 22115189 10.1186/1748-7188-6-26 PMC3319429

[RNA068239SHIC46] Love MI, Huber W, Anders S. 2014 Moderated estimation of fold change and dispersion for RNA-seq data with DESeq2. Genome Biol 15: 550. 25516281 10.1186/s13059-014-0550-8 PMC4302049

[RNA068239SHIC47] Lowe TM, Chan PP. 2016 tRNAscan-SE On-line: integrating search and context for analysis of transfer RNA genes. Nucleic Acids Res 44: W54–W57. 27174935 10.1093/nar/gkw413 PMC4987944

[RNA068239SHIC48] Mao X, Ma Q, Zhou C, Chen X, Zhang H, Yang J, Mao F, Lai W, Xu Y. 2014 DOOR 2.0: presenting operons and their functions through dynamic and integrated views. Nucleic Acids Res 42: D654–D659. 24214966 10.1093/nar/gkt1048 PMC3965076

[RNA068239SHIC49] Marcellin E, Behrendorff JB, Nagaraju S, De Tissera S, Segovia S, Palfreyman RW, Daniell J, Licona-Cassani C, Quek L, Speight R, 2016 Low carbon fuels and commodity chemicals from waste gases—systematic approach to understand energy metabolism in a model acetogen. Green Chem 18: 3020–3028.

[RNA068239SHIC50] Matthies D, Zhou W, Klyszejko AL, Anselmi C, Yildiz Ö, Brandt K, Müller V, Faraldo-Gómez JD, Meier T. 2014 High-resolution structure and mechanism of an F/V-hybrid rotor ring in a Na^+^-coupled ATP synthase. Nat Commun 5: 5286. 10.1038/ncomms6286 PMC4228694 25381992

[RNA068239SHIC51] Mironov AS, Gusarov I, Rafikov R, Lopez LE, Shatalin K, Kreneva RA, Perumov DA, Nudler E. 2002 Sensing small molecules by nascent RNA: a mechanism to control transcription in bacteria. Cell 111: 747–756. 12464185 10.1016/s0092-8674(02)01134-0

[RNA068239SHIC52] Narberhaus F, Waldminghaus T, Chowdhury S. 2006 RNA thermometers. FEMS Microbiol Rev 30: 3–16. 16438677 10.1111/j.1574-6976.2005.004.x

[RNA068239SHIC53] Naville M, Gautheret D. 2009 Transcription attenuation in bacteria: theme and variations. Brief Funct Genomic Proteomic 8: 482–492. 19651704 10.1093/bfgp/elp025

[RNA068239SHIC54] Nawrocki EP, Eddy SR. 2013 Infernal 1.1: 100-fold faster RNA homology searches. Bioinformatics 29: 2933–2935. 24008419 10.1093/bioinformatics/btt509 PMC3810854

[RNA068239SHIC55] Nawrocki EP, Burge SW, Bateman A, Daub J, Eberhardt RY, Eddy SR, Floden EW, Gardner PP, Jones TA, Tate J, 2015 Rfam 12.0: updates to the RNA families database. Nucleic Acids Res 43: D130–D137. 25392425 10.1093/nar/gku1063 PMC4383904

[RNA068239SHIC56] Nozhevnikova AN, Kotsyurbenko OR, Simankova MV. 1994 Acetogenesis at low temperature. In Acetogenesis, pp. 416–431. Springer, New York.

[RNA068239SHIC57] Nozhevnikova AN, Simankova MV, Parshina SN, Kotsyurbenko OR. 2001 Temperature characteristics of methanogenic archaea and acetogenic bacteria isolated from cold environments. Water Sci Technol 44: 41–48. 11730135

[RNA068239SHIC58] Nudler E, Mironov AS. 2004 The riboswitch control of bacterial metabolism. Trends Biochem Sci 29: 11–17. 14729327 10.1016/j.tibs.2003.11.004

[RNA068239SHIC59] Pierce E, Xie G, Barabote RD, Saunders E, Han CS, Detter JC, Richardson P, Brettin TS, Das A, Ljungdahl LG, 2008 The complete genome sequence of *Moorella thermoacetica (f. Clostridium thermoaceticum)*. Environ Microbiol 10: 2550–2573. 18631365 10.1111/j.1462-2920.2008.01679.x PMC2575129

[RNA068239SHIC60] Poehlein A, Schmidt S, Kaster A-K, Goenrich M, Vollmers J, Thürmer A, Bertsch J, Schuchmann K, Voigt B, Hecker M, 2012 An ancient pathway combining carbon dioxide fixation with the generation and utilization of a sodium ion gradient for ATP synthesis. PLoS One 7: e33439. 22479398 10.1371/journal.pone.0033439 PMC3315566

[RNA068239SHIC61] Poehlein A, Cebulla M, Ilg MM, Bengelsdorf FR, Schiel-Bengelsdorf B, Whited G, Andreesen JR, Gottschalk G, Daniel R, Dürre P. 2015 The complete genome sequence of *Clostridium aceticum*: a missing link between Rnf- and cytochrome-containing autotrophic acetogens. mBio 6: e01168-15. 26350967 10.1128/mBio.01168-15 PMC4600107

[RNA068239SHIC62] Qi L, Yue L, Feng D, Qi F, Li J, Dong X. 2017 Genome-wide mRNA processing in methanogenic archaea reveals post-transcriptional regulation of ribosomal protein synthesis. Nucleic Acids Res 45: 7285–7298. 28520982 10.1093/nar/gkx454 PMC5499594

[RNA068239SHIC63] Rach EA, Yuan H-Y, Majoros WH, Tomancak P, Ohler U. 2009 Motif composition, conservation and condition-specificity of single and alternative transcription start sites in the *Drosophila* genome. Genome Biol 10: R73. 19589141 10.1186/gb-2009-10-7-r73 PMC2728527

[RNA068239SHIC64] Regulski EE, Moy RH, Weinberg Z, Barrick JE, Yao Z, Ruzzo WL, Breaker RR. 2008 A widespread riboswitch candidate that controls bacterial genes involved in molybdenum cofactor and tungsten cofactor metabolism. Mol Microbiol 68: 918–932. 18363797 10.1111/j.1365-2958.2008.06208.x PMC2408646

[RNA068239SHIC65] Richter M, Rosselló-Móra R, Oliver Glöckner F, Peplies J. 2016 JSpeciesWS: a web server for prokaryotic species circumscription based on pairwise genome comparison. Bioinformatics 32: 929–931. 26576653 10.1093/bioinformatics/btv681 PMC5939971

[RNA068239SHIC66] Sattley WM, Madigan MT. 2007 Cold-active acetogenic bacteria from surficial sediments of perennially ice-covered Lake Fryxell, Antarctica. FEMS Microbiol Lett 272: 48–54. 17456187 10.1111/j.1574-6968.2007.00737.x

[RNA068239SHIC67] Schiel-Bengelsdorf B, Dürre P. 2012 Pathway engineering and synthetic biology using acetogens. FEBS Lett 586: 2191–2198. 22710156 10.1016/j.febslet.2012.04.043

[RNA068239SHIC68] Schuchmann K, Müller V. 2013 Direct and reversible hydrogenation of CO_2_ to formate by a bacterial carbon dioxide reductase. Science 342: 1382–1385. 24337298 10.1126/science.1244758

[RNA068239SHIC69] Schuchmann K, Müller V. 2014 Autotrophy at the thermodynamic limit of life: a model for energy conservation in acetogenic bacteria. Nat Rev Microbiol 12: 809–821. 25383604 10.1038/nrmicro3365

[RNA068239SHIC70] Sharma CM, Vogel J. 2014 Differential RNA-seq: the approach behind and the biological insight gained. Curr Opin Microbiol 19: 97–105. 25024085 10.1016/j.mib.2014.06.010

[RNA068239SHIC71] Sharma CM, Hoffmann S, Darfeuille F, Reignier J, Findeiß S, Sittka A, Chabas S, Reiche K, Hackermüller J, Reinhardt R, 2010 The primary transcriptome of the major human pathogen *Helicobacter pylori*. Nature 464: 250–255. 20164839 10.1038/nature08756

[RNA068239SHIC72] Shin J, Song Y, Jeong Y, Cho BK. 2016 Analysis of the core genome and pan-genome of autotrophic acetogenic bacteria. Front Microbiol 7: 1531. 27733845 10.3389/fmicb.2016.01531 PMC5039349

[RNA068239SHIC73] Simankova MV, Kotsyurbenko OR, Stackebrandt E, Kostrikina NA, Lysenko AM, Osipov GA, Nozhevnikova AN. 2000 *Acetobacterium tundrae* sp. nov., a new psychrophilic acetogenic bacterium from tundra soil. Arch Microbiol 174: 440–447. 11195100 10.1007/s002030000229

[RNA068239SHIC74] Singh N, Wade JT. 2014 Identification of regulatory RNA in bacterial genomes by genome-scale mapping of transcription start sites. Methods Mol Biol 1103: 1–10. 24318882 10.1007/978-1-62703-730-3_1

[RNA068239SHIC75] Sullivan MJ, Petty NK, Beatson SA. 2011 Easyfig: a genome comparison visualizer. Bioinformatics 27: 1009–1010. 21278367 10.1093/bioinformatics/btr039 PMC3065679

[RNA068239SHIC76] Tamura K, Stecher G, Peterson D, Filipski A, Kumar S. 2013 MEGA6: molecular evolutionary genetics analysis version 6.0. Mol Biol Evol 30: 2725–2729. 24132122 10.1093/molbev/mst197 PMC3840312

[RNA068239SHIC77] Tan Y, Liu J, Chen X, Zheng H, Li F. 2013 RNA-seq-based comparative transcriptome analysis of the syngas-utilizing bacterium *Clostridium ljungdahlii* DSM 13528 grown autotrophically and heterotrophically. Mol Biosyst 9: 2775–2784. 24056499 10.1039/c3mb70232d

[RNA068239SHIC78] UniProt Consortium. 2007 The Universal Protein Resource (UniProt). Nucleic Acids Res 36: D190–D195. 18045787 10.1093/nar/gkm895 PMC2238893

[RNA068239SHIC79] Vitreschak AG, Rodionov DA, Mironov AA, Gelfand MS. 2003 Regulation of the vitamin B12 metabolism and transport in bacteria by a conserved RNA structural element. RNA 9: 1084–1097. 12923257 10.1261/rna.5710303 PMC1370473

[RNA068239SHIC80] Weghoff MC, Bertsch J, Müller V. 2015 A novel mode of lactate metabolism in strictly anaerobic bacteria. Environ Microbiol 17: 670–677. 24762045 10.1111/1462-2920.12493

[RNA068239SHIC81] Wouters JA, Kamphuis HH, Hugenholtz J, Kuipers OP, de Vos WM, Abee T. 2000 Changes in glycolytic activity of *Lactococcus lactis* induced by low temperature. Appl Environ Microbiol 66: 3686–3691. 10966377 10.1128/aem.66.9.3686-3691.2000 PMC92207

