# Supplementary material for: Genome-scale analysis of Acetobacterium bakii reveals the cold adaptation of psychrotolerant acetogens by post-transcriptional regulation
Source: RNA. 2018 Dec;24(12):1839–55. doi: 10.1261/rna.068239.118 (PMC6239172; doi:10.1261/rna.068239.118)
Supplement: Supplemental Material [file supp_24_12_1839__index.html]

Genome-scale analysis of Acetobacterium bakii reveals the cold adaptation of psychrotolerant acetogens by post-transcriptional regulation — Supplemental Material 

# Genome-scale analysis of *Acetobacterium bakii* reveals the cold adaptation of psychrotolerant acetogens by post-transcriptional regulation

## Supplemental Material

- Supplemental\_Figure\_S1.pdf
- Supplemental\_Figure\_S2.pdf
- Supplemental\_Figure\_S3.pdf
- Supplemental\_Figure\_S4.pdf
- Supplemental\_Figure\_S5.pdf
- Supplemental\_Figure\_S6.pdf
- Supplemental\_Figure\_S7.pdf
- Supplemental\_Legends.docx
- Supplemental\_Table\_S1.pdf
- Supplemental\_Table\_S2.pdf
- Supplemental\_Table\_S3.pdf
- Supplemental\_Table\_S4.xlsx
- Supplemental\_Table\_S5.pdf
- Supplemental\_Table\_S6.xlsx
- Supplemental\_Table\_S7.xlsx
- Supplemental\_Table\_S8.pdf
- Supplemental\_Table\_S9.pdf
- Supplemental\_Table\_S10.pdf
