# Supplementary material for: Genome-scale analysis of Acetobacterium bakii reveals the cold adaptation of psychrotolerant acetogens by post-transcriptional regulation
Source: RNA. 2018 Dec;24(12):1839–55. doi: 10.1261/rna.068239.118 (PMC6239172; doi:10.1261/rna.068239.118)
Supplement: Supplemental Material [file supp_068239.118_Supplemental_Figure_S1.pdf]

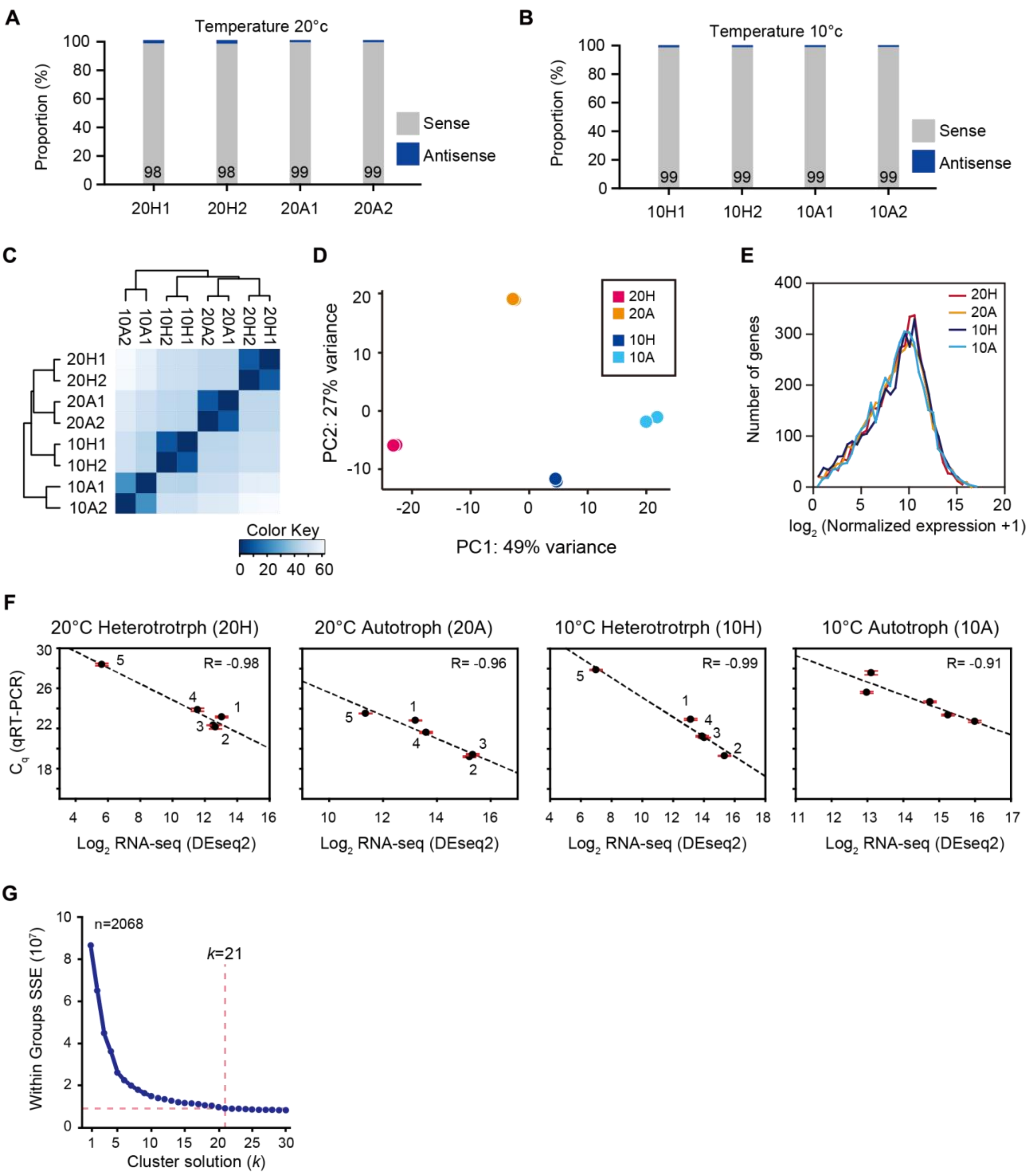

Figure S1. Reproducibility and quality of ssRNA-seq data. (**A–B**) Strand-specificity of ssRNA-seq data at (**A**) optimal temperature (20 °C) and (**B**) low temperature (10 °C). (**C**) Relationship of ssRNA-seq data among the data sets. Heat map of the sample-to-sample distance matrix is shown with hierarchical clustering using rlog-transformed read counts. (**D**) Principal component analysis (PCA) of ssRNA data. (**E**) Distribution of normalized mRNA transcript level among the four growth conditions. (**F**) Concordance between RNA-seq and qRT-PCR data. qRT-PCR was performed using RNA samples extracted independently from cultures grown in the same condition. The relationship between the normalized expression value from ssRNA-seq and the Cq value from qRT-PCR was analyzed for five genes. All plots show that the expression pattern is similar between ssRNA-seq and qRT-PCR data. Selected genes are represented by number: 1, ABAKI_c25770 encoding protein translocase subunit *secA*; 2, ABAKI_c13120 encoding corrinoid/iron-sulfur protein, small subunit *acsD* (from carbonyl-branch of WLP); 3, ABAKI_c24840 encoding methylenetetrahydrofolate reductase large subunit *metF*

(from the methyl-branch of the WLP); 4, ABAKI_c09070 encoding iron hydrogenase *hydA2* (Formate dehydrogenase cluster); 5, ABAKI_c24310 encoding electron transfer flavoprotein beta-subunit *etfB* (lactate dehydrogenase operon). (**G**) Error Sum of Squares (SSE) plot for estimating the appropriate K-means cluster number. SSE, which is defined as the sum of the squared distance among data points, was calculated by the following equation.

$$SSE = \sum_{j=1}^{p} \sum_{t=1}^{k} \sum_{i \in C_t} \left(x_{ij} - \bar{x}_j^{(t)}\right)^2$$

Based on the SSE plot, a local minimum (elbow at the 21 cluster solution) of SSE was used as the clustering number parameter. Two thousand sixty-eight differentially expressed genes (listed in Supplemental Table S4) were used in clustering analysis.
