# Supplementary material for: Genome-scale analysis of Acetobacterium bakii reveals the cold adaptation of psychrotolerant acetogens by post-transcriptional regulation
Source: RNA. 2018 Dec;24(12):1839–55. doi: 10.1261/rna.068239.118 (PMC6239172; doi:10.1261/rna.068239.118)
Supplement: Supplemental Material [file supp_068239.118_Supplemental_Figure_S2.pdf]

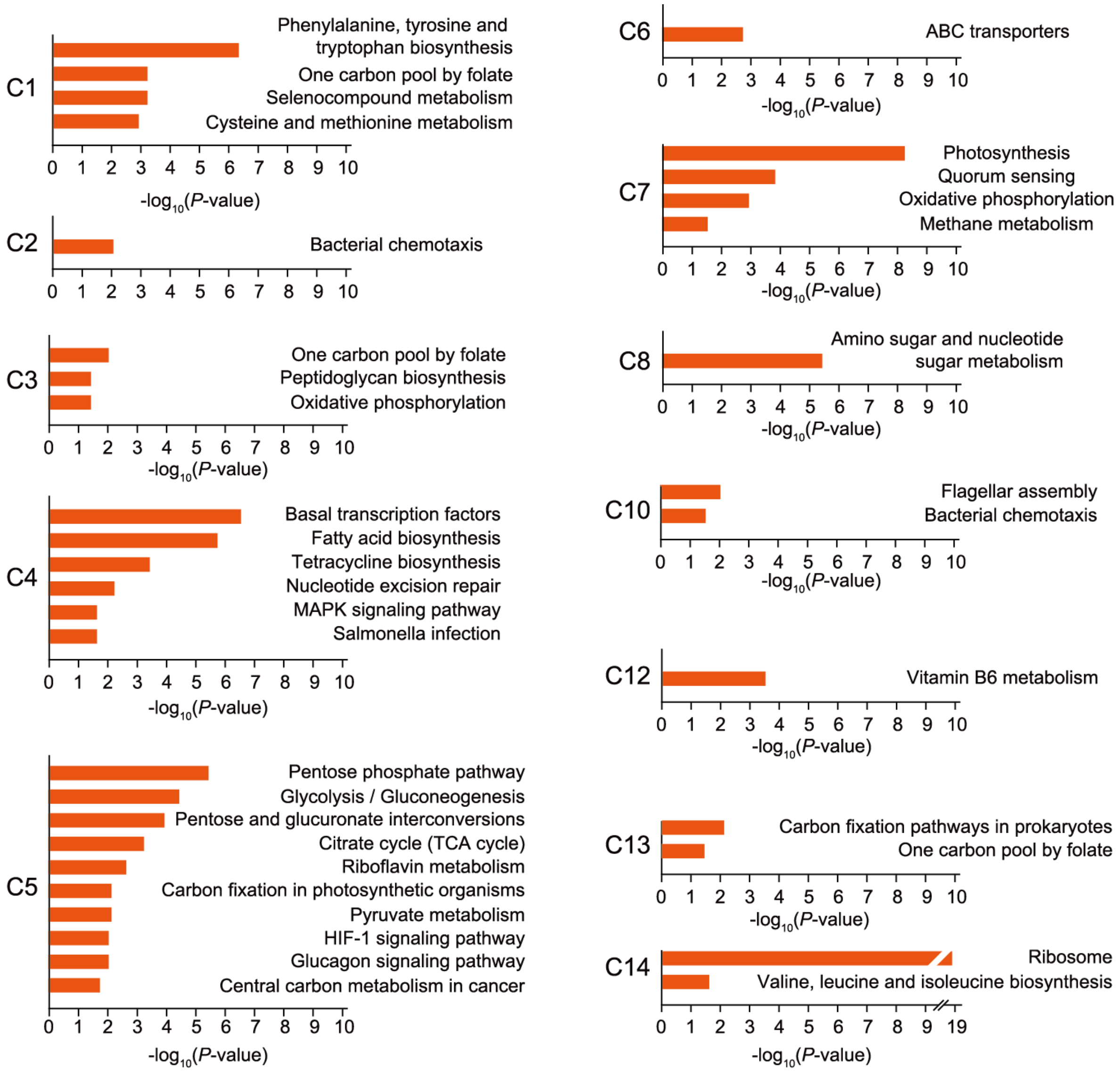


**Figure S2**. **KEGG pathways enriched in the differentially expressed genes.** The KEGG pathway enrichment analysis was performed using the groups of clustered genes. Each group was adapted to pathway enrichment analysis based on KEGG by ClueGo version 2.2.4. Bonferroni-corrected value of $P < 0.05$ was considered the cut-off criterion, and the KEGG pathways enriched in each group are shown in $-\log_{10}$(*P*-value). KEGG pathways were not enriched for the C9 and C11 groups.
