# Supplementary material for: Genome-scale analysis of Acetobacterium bakii reveals the cold adaptation of psychrotolerant acetogens by post-transcriptional regulation
Source: RNA. 2018 Dec;24(12):1839–55. doi: 10.1261/rna.068239.118 (PMC6239172; doi:10.1261/rna.068239.118)
Supplement: Supplemental Material [file supp_068239.118_Supplemental_Figure_S3.pdf]

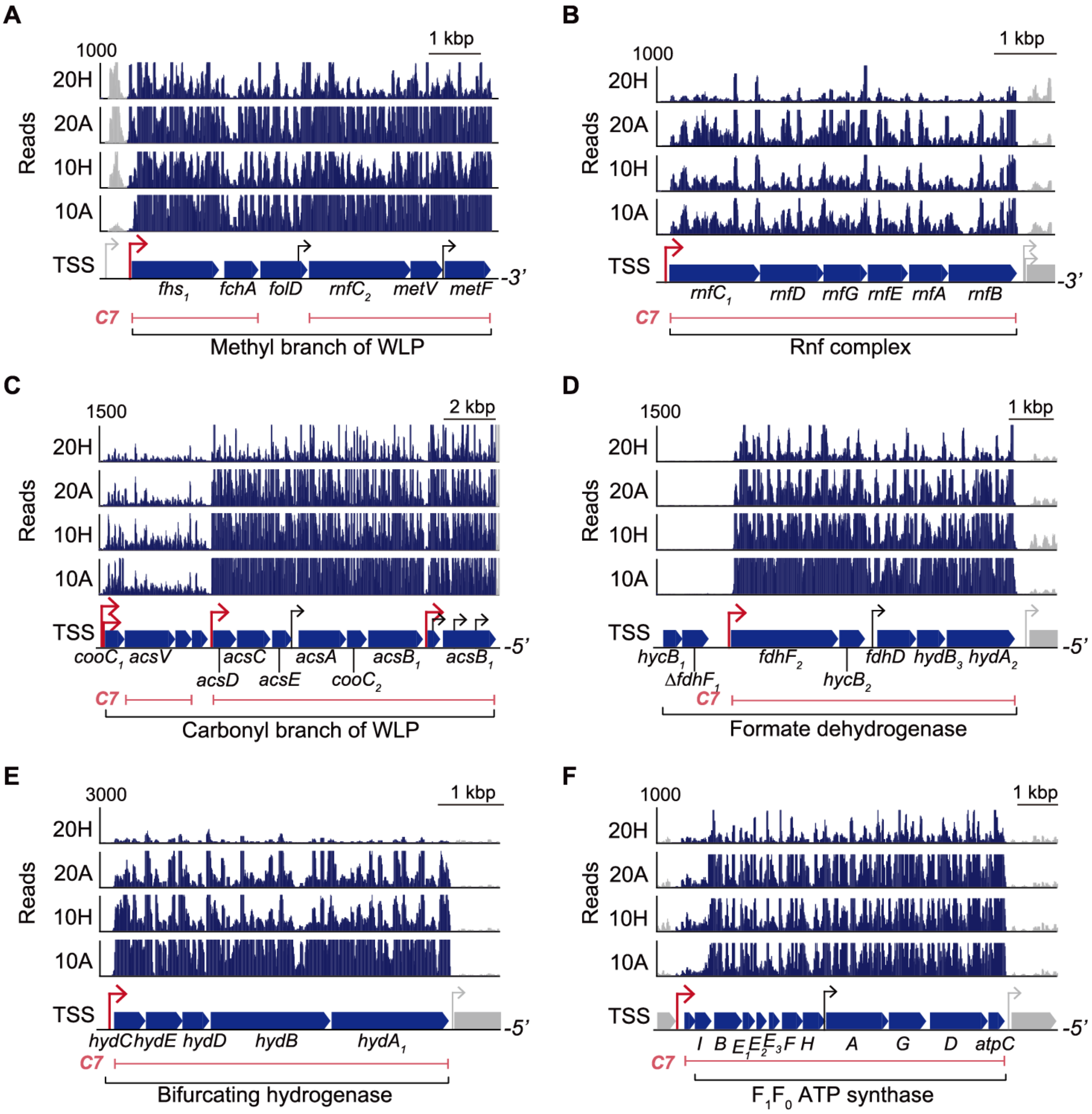

Figure S3. **Transcription units associated with acetogenesis.** (**A**) The methyl branch of the WLP (ABAKI_c24790–c24840), (**B**) the Rnf complex (ABAKI_c29390–c29440), (**C**) the carbonyl branch of the WLP (ABAKI_c13050–c13160), (**D**) the formate dehydrogenase gene cluster (ABAKI_c09070–c09130), (**E**) bifurcating hydrogenase (ABAKI_c05970–c06010), and (**F**) F_1F_0 ATP synthase (ABAKI_c18330–c18440) are shown. TSSs are illustrated as arrows, and red arrows highlight the constitutive and conditional TSSs. Red bars indicate genes in the C7 group, which are highly expressed under cold and autotrophic conditions.
