# Supplementary material for: Genome-scale analysis of Acetobacterium bakii reveals the cold adaptation of psychrotolerant acetogens by post-transcriptional regulation
Source: RNA. 2018 Dec;24(12):1839–55. doi: 10.1261/rna.068239.118 (PMC6239172; doi:10.1261/rna.068239.118)
Supplement: Supplemental Material [file supp_068239.118_Supplemental_Figure_S4.pdf]

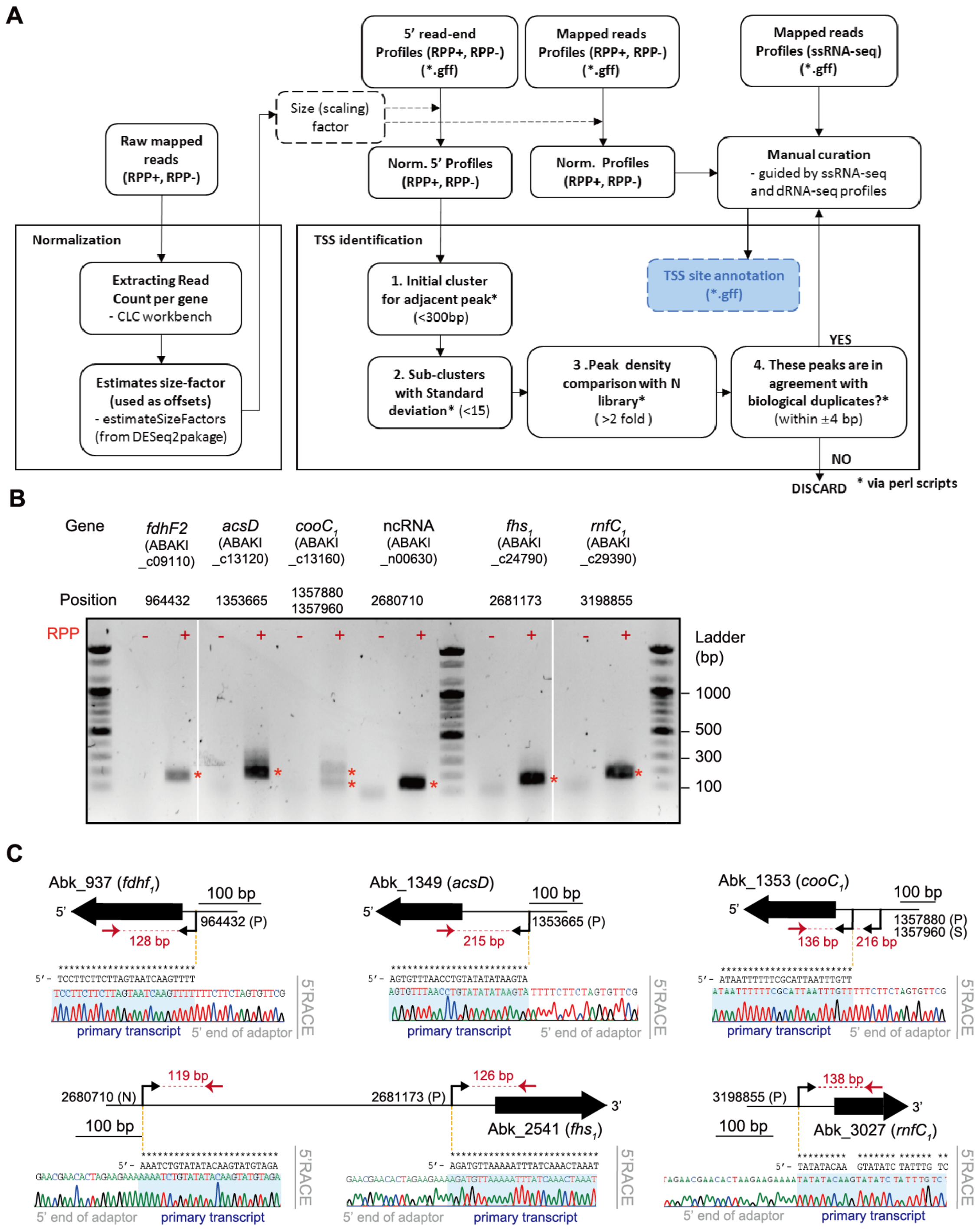

Figure S4. TSS identification and 5' Rapid Amplification of cDNA Ends (5'RACE) confirmation. (**A**) Workflow showing the key steps of TSS identification. Related to Figure 5 and Experimental Procedures. (**B**) The 2% agarose gel shows 5'RACE products. Predicted PCR bands are indicated by red asterisks. The TSSs of 6 primary mRNAs which were identified by dRNA-seq were confirmed. (**C**) Sequences of 5' RACE products were confirmed using Sanger sequencing. Black and red arrows indicate TSSs and the primers used for the amplification, respectively.
