# Supplementary material for: Genome-scale analysis of Acetobacterium bakii reveals the cold adaptation of psychrotolerant acetogens by post-transcriptional regulation
Source: RNA. 2018 Dec;24(12):1839–55. doi: 10.1261/rna.068239.118 (PMC6239172; doi:10.1261/rna.068239.118)
Supplement: Supplemental Material [file supp_068239.118_Supplemental_Figure_S5.pdf]

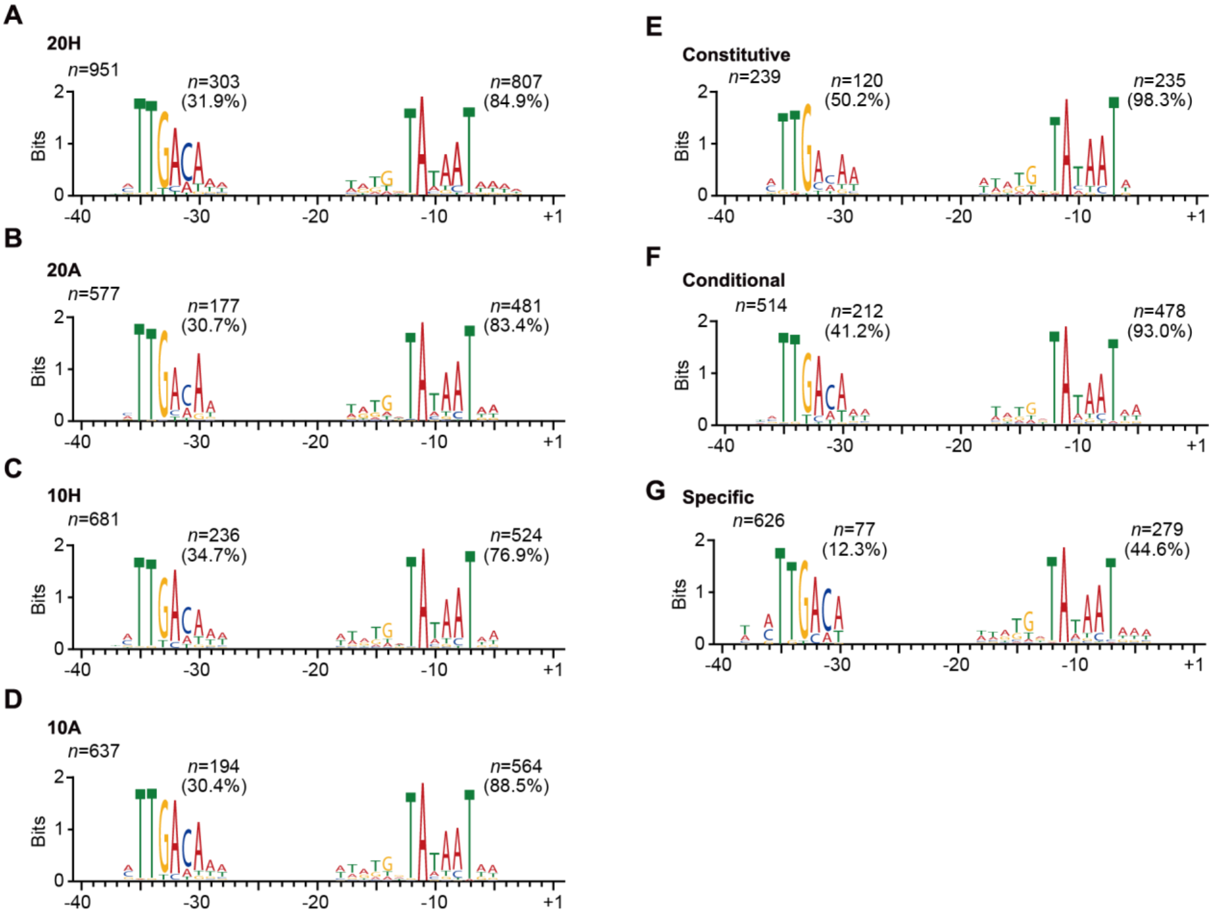

H

Gene clusters	Genes	Promoter sequences (-35 … -10)	TSS
Formate dehydrogease	ABAKI_c09110 (*fdhf*$_2$)	ACGGAGATCTGTTTGACAATACACACAACCATATGACATAATTAGGACAA	964432(P)
Carbonyl-branch of WLP	ABAKI_c13160 (*cooC*$_1$)	ACAAAATTTTCCTGTTGTAAGTTCATATGTTATAACTTAAACTGGATTAT	1357880(P)
		AAAGAATGCACTATTGTAAAGCAAGAATGATTATGGTATGATGTGCTTGT	1357960(S)
	ABAKI_c13120 (*acsD*)	ACCTGAAAAATAATTGACAATCTTTTAACTAGTATAGTACAATCTGTGTA	1353665(P)
Methyl-branch of WLP	ABAKI_c24790 (*fhs*$_1$)	TGTCACATTAAATATTTGATTTTTATTACAAAATCATTTATCATAAACGT	2681173(P)
Rnf complex	ABAKI_c29390 (*rnfC*$_1$)	TTTTCTAATGGTATAGAAAAAAAATAAAATTTATGTTATGATACTGCCCT	3198855(P)
F_1F_0 ATPsynthase	ABAKI_c18440	ATTTGTGCTGCTGTATATACATGTCTTTGAAATTGTGGTACGATATGTAA	1954708(P)
Bifurcating hydrogenase	ABAKI_c06010 (*hydC*)	TCTGTTATAAATATAACGAAATATAATGTTATGTGTTATAATTACCTCGT	640952(P)

Figure S5. Determination of promoter sequences. (**A–G**) Motif searches upstream of *A. bakii* TSS for each class of TSS. (**H**) TSSs and conserved nucleotides of the promoter in front of the first genes of acetogenesis-related gene clusters are represented with red color.
