# Supplementary material for: Genome-scale analysis of Acetobacterium bakii reveals the cold adaptation of psychrotolerant acetogens by post-transcriptional regulation
Source: RNA. 2018 Dec;24(12):1839–55. doi: 10.1261/rna.068239.118 (PMC6239172; doi:10.1261/rna.068239.118)
Supplement: Supplemental Material [file supp_068239.118_Supplemental_Figure_S6.pdf]

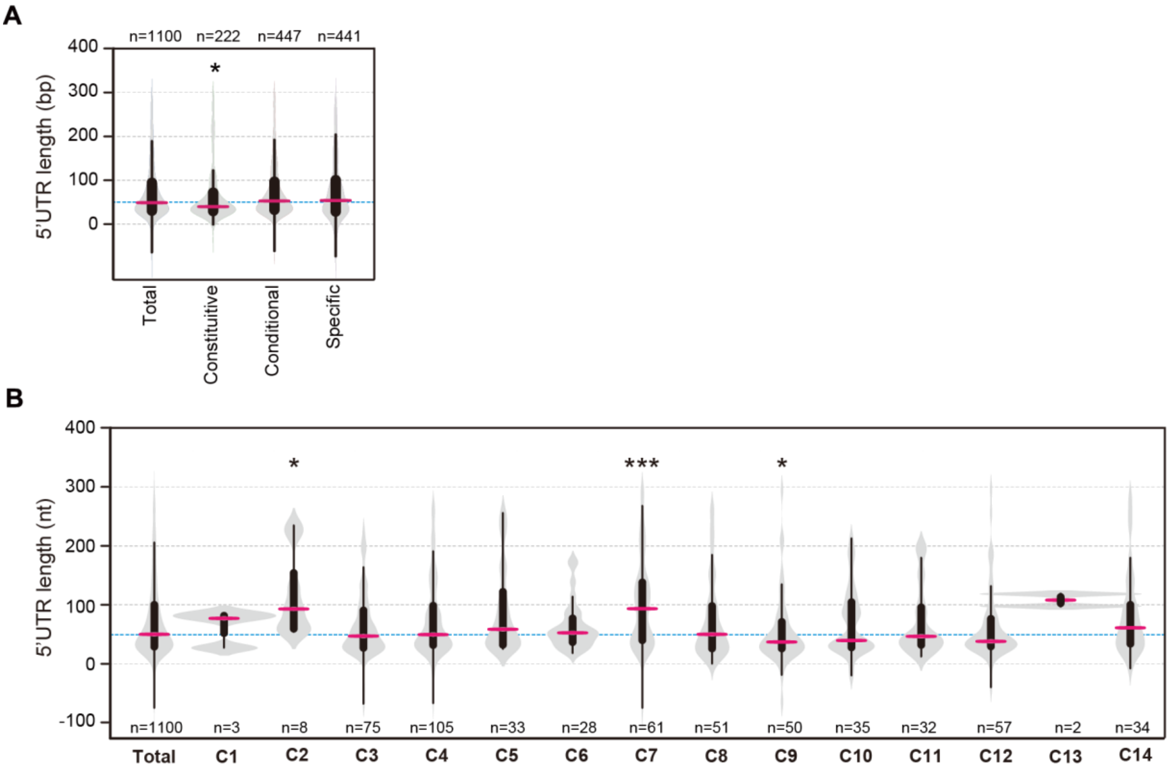

Figure S6. Comparison of the length distributions of 5'-UTRs. (**A**) Distributions of the 5'-UTR lengths defined by the total, constitutive, conditional, and specific TSSs. Constitutive primary transcripts have shorter 5'-UTRs than all primary transcripts. (**B**) Length distributions of 5' UTRs in the groups of differentially expression genes. The C2 and C7 group showed distinctly longer length distributions of 5'-UTRs, and the C9 group has shorter 5'-UTRs than all primary transcripts. The significance of differences was assessed by Wilcoxon–Mann–Whitney test (*$P < 0.05$, ***$P < 0.001$).
