# Supplementary material for: Genome-scale analysis of Acetobacterium bakii reveals the cold adaptation of psychrotolerant acetogens by post-transcriptional regulation
Source: RNA. 2018 Dec;24(12):1839–55. doi: 10.1261/rna.068239.118 (PMC6239172; doi:10.1261/rna.068239.118)
Supplement: Supplemental Material [file supp_068239.118_Supplemental_Figure_S7.pdf]

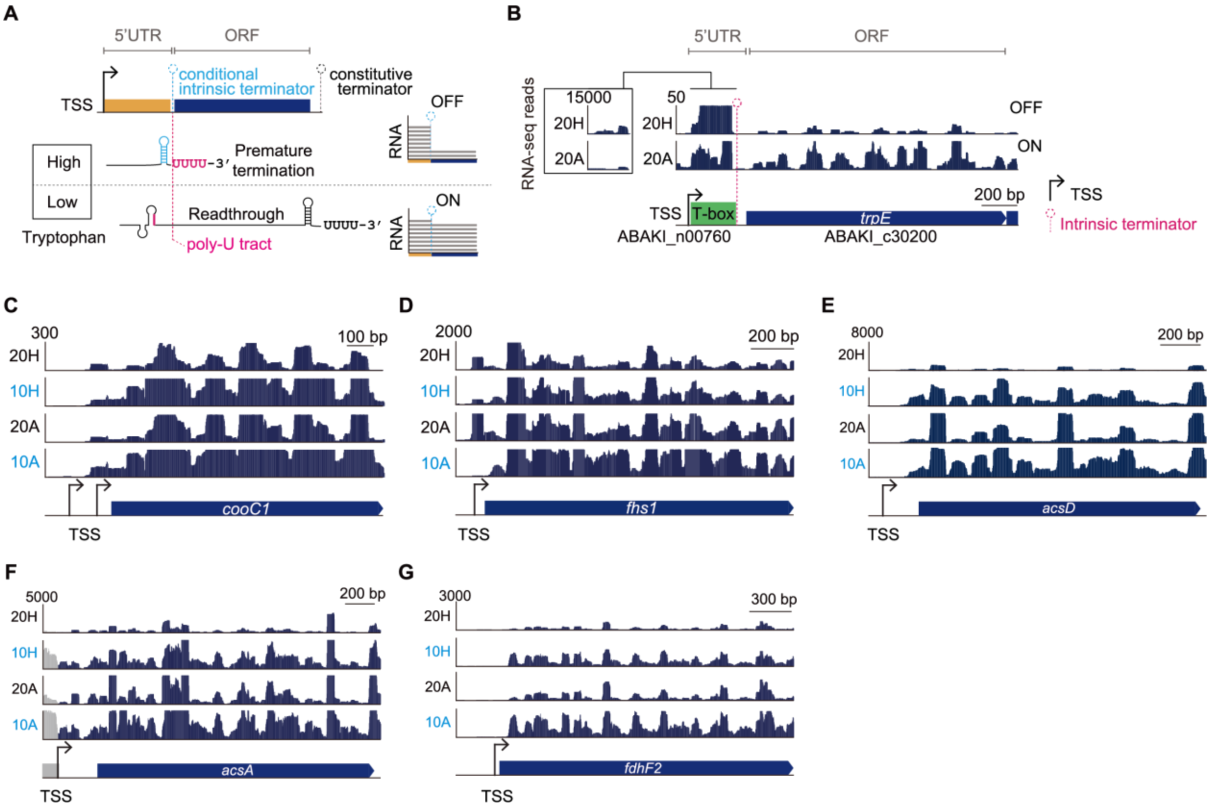

Figure S7. Transcriptional regulation by conditional termination. (**A**) A model showed 5'-UTRs containing an intrinsic terminator that differentially folded to generate a condition-specific premature termination. (**B**) A typical tryptophan riboswitch shows a typical signature of prematurely aborted termination in a 5'-UTR. (**C**) Effect of temperature on the expression of the *cooC1* gene encoding CO dehydrogenase nickel-insertion accessory protein (ABAKI_c13160), (**D**) the *fhs1* gene encoding formate--tetrahydrofolate ligase (ABAKI_c23810), (**E**) Corrinoid/iron-sulfur protein, small subunit *acsD* (ABAKI_c13120), (**F**) CO dehydrogenase, catalytic subunit *acsA* (ABAKI_c13090), and (**G**) Formate dehydrogenase H *fdhF2* (ABAKI_c09110).
