# Supplementary material for: Genome-scale analysis of Acetobacterium bakii reveals the cold adaptation of psychrotolerant acetogens by post-transcriptional regulation
Source: RNA. 2018 Dec;24(12):1839–55. doi: 10.1261/rna.068239.118 (PMC6239172; doi:10.1261/rna.068239.118)
Supplement: Supplemental Material [file supp_068239.118_Supplemental_Legends.docx]

**SUPPLEMENTARY MATERIAL**

Supplemental Figure S1 (.pdf): Reproducibility and quality of ssRNA-seq data.

Supplemental Figure S2 (.pdf): KEGG pathways enriched in the differentially expressed genes.

Supplemental Figure S3 (.pdf): Transcription units associated with acetogenesis.

Supplemental Figure S4 (.pdf): TSS identification and 5′ Rapid Amplification of cDNA Ends (5′RACE) confirmation.

Supplemental Figure S5 (.pdf): Determination of promoter sequences.

Supplemental Figure S6 (.pdf): Comparison of the length distributions of 5′-UTRs.

Supplemental Figure S7 (.pdf): Transcriptional regulation by conditional termination.

Supplemental Table S1 (.pdf): General features of the *Acetobacterium bakii* DSM 8239 genome.

Supplemental Table S2 (.pdf): Correction of sequence conflicts between short reads and PacBio scaffolds.

Supplemental Table S3 (.pdf): Statistics of RNA-Seq and dRNA-Seq analyses.

Supplemental Table S4 (.xlsx): mRNA expression profiles upon exposure to the different growth conditions.

Supplemental Table S5 (.pdf): mRNA expression profile of genes of the acetogenesis, glycolysis, and gluconeogenesis pathways.

Supplemental Table S6 (.xlsx): Transcription start sites in *A. bakii*.

Supplemental Table S7 (.xlsx): Operons of *Acetobacterium bakii.*

Supplemental Table S8 (.pdf): Predicted non-coding RNAs.

Supplemental Table S9 (.pdf): Attenuators identified in *A. bakii* and their respective mRNAs.

Supplemental Table S10 (.pdf): Oligonucleotides and DNA fragments used in this study.
