# Supplementary material for: Genome-scale analysis of Acetobacterium bakii reveals the cold adaptation of psychrotolerant acetogens by post-transcriptional regulation
Source: RNA. 2018 Dec;24(12):1839–55. doi: 10.1261/rna.068239.118 (PMC6239172; doi:10.1261/rna.068239.118)
Supplement: Supplemental Material [file supp_068239.118_Supplemental_Table_S1.pdf]

Table S1. General features of the *Acetobacterium bakii* DSM 8239 genome. (Taxonomy ID: 52689)

Feature	This study	Hwang. et al (2015)
**Scaffolds**	2<br>(4,284,306 and 34,875 bp)	90<br>(Scaffold N50: 103,215 bp)
**Genome size**	4,319,181 bp	4,135,218 bp
**GC content (%)**	41.3	41.2
**Total genes**	4151 (100.0%)	3843 (100%)
**rRNA/tRNA/ncRNA**	22/69/87 (4.3%)	5/47/1 (1.4%)
**Protein encoding sequences**	3973 (95.7%)	3718 (96.7%)
**Genes with Pfam domains**	3327 (80.1%)	3153 (82.0%)
**Genes with signal peptides**	163 (3.9%)	153 (4.0%)
**CRISPR repeat arrays**	1	1
**Accession number**	ERS1833159	NZ_LGYO00000000.1
