# Supplementary material for: Genome-scale analysis of Acetobacterium bakii reveals the cold adaptation of psychrotolerant acetogens by post-transcriptional regulation
Source: RNA. 2018 Dec;24(12):1839–55. doi: 10.1261/rna.068239.118 (PMC6239172; doi:10.1261/rna.068239.118)
Supplement: Supplemental Material [file supp_068239.118_Supplemental_Table_S2.pdf]

Table S2. Correction of sequence conflicts between short reads and PacBio scaffolds.

Chromosome	Position	Type	PacBio			MiSeq			RNA-Seq			Decesion	Polished Sequences
			5' (6 bp)		3' (6 bp)	5' (6 bp)		3' (6 bp)	5' (6 bp)		3' (6 bp)		
Scaffold1	188775	Insertion	TAGTTA	T	TTTTTT	TAGTTA	-	TTTTTT	TAGTTA	-	TTTTTT	Illumina	TAGTTATTTTTT
Scaffold1	267282	Insertion	TACAAG	T	TTTTTT	TACAAG	-	TTTTTT		n.d.		Illumina	TACAAGTTTTTT
Scaffold1	269552	Insertion	TTTTTC	A	AAAAAA	TTTTTC	-	AAAAAA	TTTTTC	-	AAAAAA	Illumina	TTTTTCAAAAAA
Scaffold1	341759	Insertion	GCCATA	T	TTTTTT	GCCATA	-	TTTTTT	GCCATA	-	TTTTTT	Illumina	GCCATATTTTTT
Scaffold1	401303	Insertion	TCCCAG	T	TTTTTT	TCCCAG	-	TTTTTT	TCCCAG	-	TTTTTT	Illumina	TCCCAGTTTTTT
Scaffold1	414296	Insertion	AATTAC	A	AAAAAA	AATTAC	-	AAAAAA		n.d.		Illumina	AATTACAAAAAA
Scaffold1	450021	Insertion	CCTATA	T	TTTTTT	CCTATA	-	TTTTTT		n.d.		Illumina	CCTATATTTTTT
Scaffold1	584056	Insertion	ATTCTA	T	TTTTTT	ATTCTA	-	TTTTTT	ATTCTA	-	TTTTTT	Illumina	ATTCTATTTTTT
Scaffold1	661591	Insertion	CATACA	T	TTTTTT	CATACA	-	TTTTTT		n.d.		Illumina	CATACATTTTTT
Scaffold1	674769	Insertion	CTCCTG	T	TTTTTT	CTCCTG	-	TTTTTT		n.d.		Illumina	CTCCTGTTTTTT
Scaffold1	679855	Insertion	TTGGGA	T	TTTTTT	TTGGGA	-	TTTTTT		n.d.		Illumina	TTGGGATTTTTT
Scaffold1	706235	Insertion	ACCGTC	A	AAAAAA	ACCGTC	-	AAAAAA	ACCGTC	-	AAAAAA	Illumina	ACCGTCAAAAAA
Scaffold1	760759	Insertion	ATGATG	T	TTTTTT	ATGATG	-	TTTTTT	ATGATG	-	TTTTTT	Illumina	ATGATGTTTTTT
Scaffold1	771530	Insertion	TATTGG	T	TTTTTT	TATTGG	-	TTTTTT	TATTGG	-	TTTTTT	Illumina	TATTGGTTTTTT
Scaffold1	789713	Insertion	TTTTTC	A	AAAAAA	TTTTTC	-	AAAAAA		n.d.		Illumina	TTTTTCAAAAAA
Scaffold1	795730	Insertion	CACCTG	T	TTTTTT	CACCTG	-	TTTTTT	CACCTG	-	TTTTTT	Illumina	CACCTGTTTTTT
Scaffold1	820820	Insertion	CATTTG	T	TTTTTT	CATTTG	-	TTTTTT		n.d.		Illumina	CATTTGTTTTTT
Scaffold1	866120	Insertion	CTTAAC	A	AAAAAA	CTTAAC	-	AAAAAA	CTTAAC	-	AAAAAA	Illumina	CTTAACAAAAAA
Scaffold1	896689	Insertion	CAACTG	T	TTTTTT	CAACTG	-	TTTTTT		n.d.		Illumina	CAACTGTTTTTT
Scaffold1	957133	Insertion	ACAGGC	T	TTTTTT	ACAGGC	-	TTTTTT	ACAGGC	-	TTTTTT	Illumina	ACAGGCTTTTTT
Scaffold1	980477	Insertion	AATTGA	T	TTTTTT	AATTGA	-	TTTTTT	AATTGA	-	TTTTTT	Illumina	AATTGATTTTTT
Scaffold1	997783	Insertion	TCTGCA	T	TTTTTT	TCTGCA	-	TTTTTT	TCTGCA	-	TTTTTT	Illumina	TCTGCATTTTTT
Scaffold1	999848	Insertion	CAACAC	A	AAAAAA	CAACAC	-	AAAAAA		n.d.		Illumina	CAACACAAAAAA
Scaffold1	1018908	Insertion	AGTGTC	A	AAAAAA	AGTGTC	-	AAAAAA	AGTGTC	-	AAAAAA	Illumina	AGTGTCAAAAAA
Scaffold1	1057372	Insertion	GCTTCC	A	AAAAAA	GCTTCC	-	AAAAAA		n.d.		Illumina	GCTTCCAAAAAA
Scaffold1	1161713	Insertion	GGCCGG	T	TTTTTT	GGCCGG	-	TTTTTT	GGCCGG	-	TTTTTT	Illumina	GGCCGGTTTTTT
Scaffold1	1178209	Insertion	CAACTG	T	TTTTTT	CAACTG	-	TTTTTT		n.d.		Illumina	CAACTGTTTTTT
Scaffold1	1266356	Insertion	ATTTGC	A	AAAAAA	ATTTGC	-	AAAAAA		n.d.		Illumina	ATTTGCAAAAAA
Scaffold1	1310361	Insertion	AACTGG	T	TTTTTT	AACTGG	-	TTTTTT		n.d.		Illumina	AACTGGTTTTTT
Scaffold1	1381255	Insertion	TTTAAT	A	AAAAAA	TTTAAT	-	AAAAAA		n.d.		Illumina	TTTAATAAAAAA
Scaffold1	1413294	Insertion	TAATGC	A	AAAAAA	TAATGC	-	AAAAAA		n.d.		Illumina	TAATGCAAAAAA
Scaffold1	1440220	Insertion	CATCTG	T	TTTTTT	CATCTG	-	TTTTTT	CATCTG	-	TTTTTT	Illumina	CATCTGTTTTTT
Scaffold1	1448959	Insertion	CCCACC	A	AAAAAA	CCCACC	-	AAAAAA	CCCACC	-	AAAAAA	Illumina	CCCACCAAAAAA
Scaffold1	1491169	Insertion	ACGTCG	T	TTTTTT	ACGTCG	-	TTTTTT	ACGTCG	-	TTTTTT	Illumina	ACGTCGTTTTTT
Scaffold1	1568251	Insertion	ATAGTC	A	AAAAAA	ATAGTC	-	AAAAAA	ATAGTC	-	AAAAAA	Illumina	ATAGTCAAAAAA
Scaffold1	1612865	Insertion	TGTATA	T	TTTTTT	TGTATA	-	TTTTTT	TGTATA	-	TTTTTT	Illumina	TGTATATTTTTT
Scaffold1	1627657	Insertion	TCACTG	T	TTTTTT	TCACTG	-	TTTTTT	TCACTG	-	TTTTTT	Illumina	TCACTGTTTTTT

Scaffold1	1632066	Insertion	ACTCAG	T	TTTTTT	ACTCAG	-	TTTTTT	ACTCAG	-	TTTTTT	Illumina	ACTCAGTTTTTT
Scaffold1	1646664	Insertion	GCCATG	T	TTTTTT	GCCATG	-	TTTTTT		n.d.		Illumina	GCCATGTTTTTT
Scaffold1	1658108	Insertion	CAACTG	T	TTTTTT	CAACTG	-	TTTTTT		n.d.		Illumina	CAACTGTTTTTT
Scaffold1	1713991	Insertion	TTTTTG	T	TTTTTT	TTTTTG	-	TTTTTT	TTTTTG	-	TTTTTT	Illumina	TTTTTGTTTTTT
Scaffold1	1802627	Insertion	CCTCGG	A	AAAAAA	CCTCGG	-	AAAAAA	CCTCGG	-	AAAAAA	Illumina	CCTCGGAAAAAA
Scaffold1	1814925	Insertion	TAACAT	A	AAAAAA	TAACAT	-	AAAAAA		n.d.		Illumina	TAACATAAAAAA
Scaffold1	1896159	Insertion	CTATCA	T	TTTTTT	CTATCA	-	TTTTTT		n.d.		Illumina	CTATCATTTTTT
Scaffold1	1896832	Insertion	TTTTGA	T	TTTTTT	TTTTGA	-	TTTTTT	TTTTGA	-	TTTTTT	Illumina	TTTTGATTTTTT
Scaffold1	1909603	Insertion	ATAAGC	A	AAAAAA	ATAAGC	-	AAAAAA	ATAAGC	-	AAAAAA	Illumina	ATAAGCAAAAAA
Scaffold1	1931069	Insertion	TTTTTG	A	AAAAAA	TTTTTG	-	AAAAAA		n.d.		Illumina	TTTTTGAAAAAA
Scaffold1	1966223	Insertion	CCCGGC	A	AAAAAA	CCCGGC	-	AAAAAA	CCCGGC	-	AAAAAA	Illumina	CCCGGCAAAAAA
Scaffold1	2018777	Insertion	CACCCG	T	TTTTTT	CACCCG	-	TTTTTT		n.d.		Illumina	CACCCGTTTTTT
Scaffold1	2038641	Insertion	CCCGTC	T	TTTTTT	CCCGTC	-	TTTTTT	CCCGTC	-	TTTTTT	Illumina	CCCGTCTTTTTT
Scaffold1	2081233	SNV	TTTTTT	T	AAATCC	TTTTTT	A	AAATCC	TTTTTT	A	AAATCC	Illumina	TTTTTTAAAATCC
Scaffold1	2141103	Insertion	AAAATC	A	AAAAAA	AAAATC	-	AAAAAA	AAAATC	-	AAAAAA	Illumina	AAAATCAAAAAA
Scaffold1	2186131	Insertion	GATTTC	A	AAAAAA	GATTTC	-	AAAAAA		n.d.		Illumina	GATTTCAAAAAA
Scaffold1	2247757	Insertion	TCGCAG	T	TTTTTT	TCGCAG	-	TTTTTT	TCGCAG	-	TTTTTT	Illumina	TCGCAGTTTTTT
Scaffold1	2261363	Insertion	TCGATG	A	AAAAAA	TCGATG	-	AAAAAA	TCGATG	-	AAAAAA	Illumina	TCGATGAAAAAA
Scaffold1	2409696	Insertion	ACCTTG	T	TTTTTT	ACCTTG	-	TTTTTT	ACCTTG	-	TTTTTT	Illumina	ACCTTGTTTTTT
Scaffold1	2428716	Insertion	TTTCAC	A	AAAAAA	TTTCAC	-	AAAAAA	TTTCAC	-	AAAAAA	Illumina	TTTCACAAAAAA
Scaffold1	2452609	Insertion	CTTTGC	A	AAAAAA	CTTTGC	-	AAAAAA		n.d.		Illumina	CTTTGCAAAAAA
Scaffold1	2460317	Insertion	ATAAGG	T	TTTTTT	ATAAGG	-	TTTTTT		n.d.		Illumina	ATAAGGTTTTTT
Scaffold1	2473989	Insertion	CATCCG	A	AAAAAA	CATCCG	-	AAAAAA	CATCCG	-	AAAAAA	Illumina	CATCCGAAAAAA
Scaffold1	2500147	Insertion	ATCGGC	A	AAAAAA	ATCGGC	-	AAAAAA	ATCGGC	-	AAAAAA	Illumina	ATCGGCAAAAAA
Scaffold1	2518161	Insertion	AAAATC	A	AAAAAA	AAAATC	-	AAAAAA	AAAATC	-	AAAAAA	Illumina	AAAATCAAAAAA
Scaffold1	2535628	Insertion	TATAGT	A	AAAAAA	TATAGT	-	AAAAAA	TATAGT	-	AAAAAA	Illumina	TATAGTAAAAAA
Scaffold1	2544001	Insertion	AGTGAG	T	TTTTTT	AGTGAG	-	TTTTTT		n.d.		Illumina	AGTGAGTTTTTT
Scaffold1	2590861	Insertion	CATAGG	T	TTTTTT	CATAGG	-	TTTTTT		n.d.		Illumina	CATAGGTTTTTT
Scaffold1	2594706	Insertion	TTCTGC	A	AAAAAA	TTCTGC	-	AAAAAA	TTCTGC	-	AAAAAA	Illumina	TTCTGCAAAAAA
Scaffold1	2647823	Insertion	AGAAGC	A	AAAAAA	AGAAGC	-	AAAAAA	AGAAGC	-	AAAAAA	Illumina	AGAAGCAAAAAA
Scaffold1	2671901	Insertion	AGGTTC	A	AAAAAA	AGGTTC	-	AAAAAA		n.d.		Illumina	AGGTTCAAAAAA
Scaffold1	2705711	Insertion	AAACCG	T	TTTTTT	AAACCG	-	TTTTTT	AAACCG	-	TTTTTT	Illumina	AAACCGTTTTTT
Scaffold1	2743629	Insertion	AATTTG	A	AAAAAA	AATTTG	-	AAAAAA	AATTTG	-	AAAAAA	Illumina	AATTTGAAAAAA
Scaffold1	2746381	Insertion	AACTTC	A	AAAAAA	AACTTC	-	AAAAAA		n.d.		Illumina	AACTTCAAAAAA
Scaffold1	2814005	Insertion	AAAAAT	A	AAAAAA	AAAAAT	-	AAAAAA		n.d.		Illumina	AAAAATAAAAAA
Scaffold1	2901055	Insertion	TAGCTG	T	TTTTTT	TAGCTG	-	TTTTTT	TAGCTG	-	TTTTTT	Illumina	TAGCTGTTTTTT
Scaffold1	2930921	Insertion	CAAAGC	A	AAAAAA	CAAAGC	-	AAAAAA		n.d.		Illumina	CAAAGCAAAAAA
Scaffold1	2969605	Insertion	GTTTGA	T	TTTTTT	GTTTGA	-	TTTTTT	GTTTGA	-	TTTTTT	Illumina	GTTTGATTTTTT
Scaffold1	3012801	Insertion	TATCTG	A	AAAAAA	TATCTG	-	AAAAAA		n.d.		Illumina	TATCTGAAAAAA
Scaffold1	3204175	Insertion	ATTTTT	A	AAAAAA	ATTTTT	-	AAAAAA		n.d.		Illumina	ATTTTTAAAAAA
Scaffold1	3234138	Insertion	AGGCAG	A	AAAAAA	AGGCAG	-	AAAAAA	AGGCAG	-	AAAAAA	Illumina	AGGCAGAAAAAA

Scaffold1	3241192	Insertion	AAAATC	A	AAAAAA	AAAATC	-	AAAAAA	AAAATC	-	AAAAAA	Illumina	AAAATCAAAAAA
Scaffold1	3249433	Insertion	CAATTG	T	TTTTTT	CAATTG	-	TTTTTT	CAATTG	-	TTTTTT	Illumina	CAATTGTTTTTT
Scaffold1	3344148	Insertion	GAAGAC	A	AAAAAA	GAAGAC	-	AAAAAA		n.d.		Illumina	GAAGACAAAAAA
Scaffold1	3386305	Insertion	GATCAG	T	TTTTTT	GATCAG	-	TTTTTT		n.d.		Illumina	GATCAGTTTTTT
Scaffold1	3422712	Insertion	GCTGGG	T	TTTTTT	GCTGGG	-	TTTTTT		n.d.		Illumina	GCTGGGTTTTTT
Scaffold1	3428846	Insertion	AATGGC	A	AAAAAA	AATGGC	-	AAAAAA	AATGGC	-	AAAAAA	Illumina	AATGGCAAAAAA
Scaffold1	3484083	Insertion	TGTCTG	A	AAAAAA	TGTCTG	-	AAAAAA	TGTCTG	-	AAAAAA	Illumina	TGTCTGAAAAAA
Scaffold1	3486869	Insertion	AAGGAA	T	TTTTTT	AAGGAA	-	TTTTTT	AAGGAA	-	TTTTTT	Illumina	AAGGAATTTTTT
Scaffold1	3535985	Insertion	CAGTGC	A	AAAAAA	CAGTGC	-	AAAAAA	CAGTGC	-	AAAAAA	Illumina	CAGTGCAAAAAA
Scaffold1	3601116	Insertion	AACTGC	A	AAAAAA	AACTGC	-	AAAAAA		n.d.		Illumina	AACTGCAAAAAA
Scaffold1	3608445	Insertion	AGAACG	A	AAAAAA	AGAACG	-	AAAAAA	AGAACG	-	AAAAAA	Illumina	AGAACGAAAAAA
Scaffold1	3626509	Insertion	ACCTCG	T	TTTTTT	ACCTCG	-	TTTTTT		n.d.		Illumina	ACCTCGTTTTTT
Scaffold1	3690638	Insertion	CAGCGC	A	AAAAAA	CAGCGC	-	AAAAAA	CAGCGC	-	AAAAAA	Illumina	CAGCGCAAAAAA
Scaffold1	3703679	Insertion	GCATTG	T	TTTTTT	GCATTG	-	TTTTTT	GCATTG	-	TTTTTT	Illumina	GCATTGTTTTTT
Scaffold1	3712698	Insertion	ACATGC	A	AAAAAA	ACATGC	-	AAAAAA	ACATGC	-	AAAAAA	Illumina	ACATGCAAAAAA
Scaffold1	3741176	Insertion	CAGGTA	T	TTTTTT	CAGGTA	-	TTTTTT	CAGGTA	-	TTTTTT	Illumina	CAGGTATTTTTT
Scaffold1	3774712	Insertion	TGGCTC	A	AAAAAA	TGGCTC	-	AAAAAA		n.d.		Illumina	TGGCTCAAAAAA
Scaffold1	3784724	Insertion	GCAGGC	T	TTTTTT	GCAGGC	-	TTTTTT		n.d.		Illumina	GCAGGCTTTTTT
Scaffold1	3800864	Insertion	TTCGCG	T	TTTTTT	TTCGCG	-	TTTTTT	TTCGCG	-	TTTTTT	Illumina	TTCGCGTTTTTT
Scaffold1	3812344	Insertion	TAACAA	A	AAAAAA	TAACAA	-	AAAAAA		n.d.		Illumina	TAACAAAAAAAA
Scaffold1	3841980	Insertion	ACCGAC	A	AAAAAA	ACCGAC	-	AAAAAA		n.d.		Illumina	ACCGACAAAAAA
Scaffold1	3898871	Insertion	GACAGC	A	AAAAAA	GACAGC	-	AAAAAA	GACAGC	-	AAAAAA	Illumina	GACAGCAAAAAA
Scaffold1	3923515	Insertion	TGATAA	T	TTTTTT	TGATAA	-	TTTTTT		n.d.		Illumina	TGATAATTTTTT
Scaffold1	3939455	Insertion	ATGCTC	A	AAAAAA	ATGCTC	-	AAAAAA		n.d.		Illumina	ATGCTCAAAAAA
Scaffold1	3963348	Insertion	GTATTG	T	TTTTTT	GTATTG	-	TTTTTT	GTATTG	-	TTTTTT	Illumina	GTATTGTTTTTT
Scaffold1	3976393	Insertion	CAGTAG	T	TTTTTT	CAGTAG	-	TTTTTT	CAGTAG	-	TTTTTT	Illumina	CAGTAGTTTTTT
Scaffold1	4093008	Insertion	ATTATC	A	AAAAAA	ATTATC	-	AAAAAA	ATTATC	-	AAAAAA	Illumina	ATTATCAAAAAA
Scaffold1	4242975	Insertion	TAATTG	T	TTTTTT	TAATTG	-	TTTTTT		n.d.		Illumina	TAATTGTTTTTT
Scaffold1	4273452	SNV	ATTGAC	A	ATCATT	ATTGAC	G	ATCATT	ATTGAC	G	ATCATT	Illumina	ATTGACGATCATT
Scaffold1	4273473	SNV	TATCCG	T	ATGAAG	TATCCG	G	ATGAAG	TATCCG	G	ATGAAG	Illumina	TATCCGGATGAAG
Scaffold1	4273481	SNV	TGAAGC	C	ATTGCT	TGAAGC	T	ATTGCT	TGAAGC	T	ATTGCT	Illumina	TGAAGCTATTGCT
Scaffold1	4273498	SNV	AAATCT	A	CTTCTG	AAATCT	G	CTTCTG	AAATCT	G	CTTCTG	Illumina	AAATCTGCTTCTG
Scaffold1	4273503	SNV	TGCTTC	C	GCGTAT	TGCTTC	T	GCGTAT	TGCTTC	T	GCGTAT	Illumina	TGCTTCTGCGTAT
Scaffold1	4276589	Insertion	TTTCGC	A	AAAAAA	TTTCGC	-	AAAAAA	TTTCGC	-	AAAAAA	Illumina	TTTCGCAAAAAA
