# Supplementary material for: Genome-scale analysis of Acetobacterium bakii reveals the cold adaptation of psychrotolerant acetogens by post-transcriptional regulation
Source: RNA. 2018 Dec;24(12):1839–55. doi: 10.1261/rna.068239.118 (PMC6239172; doi:10.1261/rna.068239.118)
Supplement: Supplemental Material [file supp_068239.118_Supplemental_Table_S3.pdf]

Table S3. Statistics of ssRNA and dRNA-sequencing.

Library	Sample	Raw data		Quality and adapter trimming		Genome mapping (total)				Genome mapping (unique)			
		Reads	Length	Remaining reads	Discarded (%)	Mapped Reads	# of bases	Mapped (%)	Covarage	Mapped Reads	# of bases	Mapped (%)	Covarage
ssRNA-seq	20_H1	23,222,472	51	23,219,127	0.0	22,443,904	1,143,429,259	96.6	272	16,706,884	851,311,045	71.9	203
	20_H2	12,930,518	51	12,930,518	0.0	12,355,219	629,289,845	95.6	150	8,725,145	444,519,801	67.5	106
	20_A1	12,628,973	51	12,628,125	0.0	11,306,556	575,862,047	89.5	137	8,779,865	447,309,916	69.5	107
	20_A2	18,353,314	51	18,351,423	0.0	15,024,813	765,471,306	81.9	182	11,382,084	580,011,966	62.0	138
	10_H1	24,873,291	51	24,871,515	0.0	24,805,671	1,260,417,394	99.7	300	23,000,473	1,168,830,462	92.5	278
	10_H2	19,208,162	51	19,206,648	0.0	19,158,062	973,512,450	99.7	232	17,797,967	904,500,249	92.7	215
	10_A1	21,135,880	51	21,135,880	0.0	21,054,344	1,069,417,581	99.6	255	17,906,149	909,775,344	84.7	217
	10_A2	22,545,745	51	22,545,745	0.0	22,474,241	1,141,976,582	99.7	272	19,752,303	1,003,884,855	87.6	239
dRNA-seq	20_H1_RPP+	7,793,136	100	7,663,947	1.7	7,514,967	751,848,764	96.4	179	7,362,095	736,624,648	94.5	175
	20_H1_RPP-	8,801,273	100	7,669,835	12.9	7,266,517	724,369,687	82.6	172	7,078,142	705,926,485	80.4	168
	20_H2_RPP+	8,092,363	100	6,733,705	16.8	6,510,319	648,486,956	80.5	154	6,393,520	637,046,066	79.0	152
	20_H2_RPP-	8,092,363	100	3,252,342	59.8	2,937,269	290,165,553	36.3	69	2,855,913	282,323,949	35.3	67
	20_A1_RPP+	8,748,019	100	7,496,773	14.3	7,232,370	720,649,895	82.7	172	7,061,533	704,153,932	80.7	168
	20_A1_RPP-	7,359,463	100	3,590,110	51.2	2,960,391	292,525,464	40.2	70	2,889,109	285,847,626	39.3	68
	20_A2_RPP+	9,822,667	100	9,247,874	5.9	8,355,445	825,492,459	85.1	197	8,154,069	806,709,405	83.0	192
	20_A2_RPP-	6,380,411	100	3,039,518	52.4	2,593,578	256,896,692	40.6	61	2,504,689	248,504,568	39.3	59
	10_H1_RPP+	13,400,573	100	12,790,336	4.6	11,251,038	1,086,530,924	84.0	259	10,885,556	1,053,057,416	81.2	251
	10_H1_RPP-	10,021,910	100	8,196,032	18.2	6,117,694	586,823,866	61.0	140	5,883,457	566,102,646	58.7	135
	10_H2_RPP+	12,758,706	100	11,150,382	12.6	7,809,449	689,119,420	61.2	164	7,393,303	660,248,506	57.9	157
	10_H2_RPP-	9,458,132	100	9,458,132	0.0	7,616,544	725,800,332	80.5	173	7,239,837	693,203,014	76.5	165

	10_A1_ RPP+	9,560,657	100	8,364,998	12.5	7,201,025	713,519,340	75.3	170	6,195,262	613,895,923	64.8	146
	10_A1_ RPP-	7,681,907	100	5,112,845	33.4	3,546,437	339,651,492	46.2	81	2,895,034	277,659,396	37.7	66
	10_A2_ RPP+	10,872,050	100	10,064,516	7.4	9,692,022	954,318,397	89.1	227	9,366,109	923,663,600	86.1	220
	10_A2_ RPP-	7,591,691	100	5,540,130	27.0	2,925,870	275,663,221	38.5	66	2,608,700	249,179,656	34.4	59
