# Supplementary material for: Genome-scale analysis of Acetobacterium bakii reveals the cold adaptation of psychrotolerant acetogens by post-transcriptional regulation
Source: RNA. 2018 Dec;24(12):1839–55. doi: 10.1261/rna.068239.118 (PMC6239172; doi:10.1261/rna.068239.118)
Supplement: Supplemental Material [file supp_068239.118_Supplemental_Table_S5.pdf]

Table S5. mRNA expression profiles of the acetogenesis, glycolysis, and gluconeogenesis pathways.

Pathway	Gene or gene clusters	Locus_tag	Normalized gene expression			
			20H	20A	10H	10A
Acetyl-CoA (Wood-Ljungdahl) pathway	Formate dehydrogenase gene cluster (fdhF2,hycB2, fdhD, hycB3, hydA2)	ABAKI_c09070	2988.9	12408.6	15158.9	27401.4
		ABAKI_c09080	683.5	3376.7	2954.9	6798.6
		ABAKI_c09090	1166.4	4041.8	5365.2	7948.5
		ABAKI_c09100	1418.7	3928.6	5108.7	13148.8
		ABAKI_c09110	6154.4	16011.3	20750.1	45514.7
	Formyl-THF synthetase (fhs1)	ABAKI_c24790	27502.6	76193.7	42083.3	74046.4
	Formyl-THF-cyclohydrolase (fchA)	ABAKI_c24800	2401.7	11318.2	5087.3	6214.8
	Methylene-THF-dehydrogenase (folD)	ABAKI_c24810	6886.1	34612.3	10369.4	11985.8
	Methylene-THF-reductase (rnfC2, metV, metF)	ABAKI_c24820	11428.4	68228.0	24595.6	58418.1
		ABAKI_c24830	3877.7	25917.0	8235.8	17598.5
		ABAKI_c24840	6438.5	41263.6	16705.7	38803.3
	Carbonyl-branch gene cluster (Acetyl-CoA synthase/CO dehydrogenase) (acsB1, cooC2, acsA, acsE, acsC, acsD, acsV cooC1)	ABAKI_c13050	28925.7	63552.2	74607.4	75126.5
		ABAKI_c13060	8664.9	18451.4	17383.0	19009.3
		ABAKI_c13070	16164.1	77870.3	79249.8	149510.8
		ABAKI_c13080	6023.9	23501.8	19195.6	32052.3
		ABAKI_c13090	17064.5	77042.2	67432.0	105650.1
		ABAKI_c13100	4244.7	29629.3	33226.3	53334.1
		ABAKI_c13110	4984.9	36951.9	60285.0	96990.0
		ABAKI_c13120	5860.3	37970.7	41515.1	65208.6
		ABAKI_c13130	733.5	1251.4	3777.9	2525.2
		ABAKI_c13140	1621.6	2668.0	5756.9	3283.0
		ABAKI_c13150	3793.2	7697.5	13838.5	11224.3
		ABAKI_c13160	1932.8	3776.9	6948.8	9545.0
Acetate fermentation pathway	Phosphate butyryltransferase (PTB)	ABAKI_c02310	989.4	1022.3	1953.8	1671.1
	Acetate kinase (ACK)	ABAKI_c22980	41.8	22.4	7.4	36.0
		ABAKI_c35350	4456.3	12856.9	15827.7	26740.9
EMP pathway	Phosphotransferase system (PTS)	ABAKI_c17240	13478.3	51608.7	15039.4	10430.6
	1-phosphofructokinase (Fruk)	ABAKI_c17250	7023.9	16648.9	5958.2	3552.4
	6-phosphofructokinase (PFK)	ABAKI_c22540	57.9	31.4	19.3	65.3
		ABAKI_c26240	3143.7	2167.1	1981.4	1923.5

		ABAKI_c36320	144.3	252.3	877.9	697.5
	Fructose-1,6-bisphosphate aldolase Fba	ABAKI_c08200	23082.7	3728.8	2621.1	2187.6
		ABAKI_c12750	2034.6	4148.2	3348.5	4764.9
		ABAKI_c25870	971.1	39.6	12.5	39.8
		ABAKI_c35620	1353.0	112.7	140.0	434.9
	Triosephosphate isomerase (TIM)	ABAKI_c26590	2817.2	3252.4	2660.1	2947.1
		ABAKI_c24360	3989.2	4504.8	2156.0	2895.9
	Glyceraldehyde-3-phosphate dehydrogenase (GAPDH)	ABAKI_c26570	10911.9	14263.7	15497.6	14351.1
	Glyceraldehyde-3-phosphate dehydrogenase, type II (GAP2)	ABAKI_c11080	280.3	156.2	132.3	1032.6
	Phosphoglycerate kinase (PKG)	ABAKI_c11070	245.9	192.4	151.9	1024.8
		ABAKI_c26580	6465.6	7562.8	5946.9	5802.9
	2,3-bisphosphoglycerate-independent phosphoglyceratemutase(iPGAM))	ABAKI_c26600	11788.3	11528.7	7109.3	7222.1
	Enolase (ENO)	ABAKI_c22260	2804.6	1672.6	2795.6	8986.7
	Pyruvate kinase (PK)	ABAKI_c26250	8458.6	6551.8	5726.6	5878.0
	Pyruvate:ferredoxin oxidoreductase (PFOR)	ABAKI_c22150	1285.9	820.4	890.0	765.5
		ABAKI_c22160	2152.5	1272.4	1711.6	2181.1
Lactate fermentation pathway	Lactate dehydrogenase (lctB, C, D, E, F)	ABAKI_c24310	49.1	2610.0	126.6	8075.1
		ABAKI_c24320	84.6	4937.6	267.5	9107.2
		ABAKI_c24330	64.8	3235.4	152.2	10909.0
		ABAKI_c24340	76.5	3976.5	164.3	9254.1
		ABAKI_c24350	32.3	702.8	46.1	2028.5
ATPsynthase	F_1F_O ATPsynthase (atpI, B, E1, E2, E3, F,H, A, G, D, C)	ABAKI_c18330	3125.5	15392.2	9966.5	12351.4
		ABAKI_c18340	6661.7	32941.5	21419.9	25235.1
		ABAKI_c18350	5276.0	25997.6	13059.7	17028.3
		ABAKI_c18360	5774.6	32286.0	18757.2	26352.6
		ABAKI_c18370	1171.4	6749.8	3145.7	4031.7
		ABAKI_c18380	1511.9	9115.8	5462.5	5242.1
		ABAKI_c18390	349.3	1826.5	1110.0	997.4
		ABAKI_c18400	253.8	1459.2	822.7	987.5
		ABAKI_c18410	834.5	4202.0	2222.8	3514.7
		ABAKI_c18420	1721.9	7716.5	5984.2	7138.2
		ABAKI_c18430	553.4	1826.0	1765.4	1471.1
Rnf complex	Rnf conplex (rnfC1, D, G, E, A, B)	ABAKI_c29390	3043.0	14933.0	7748.2	11577.9
		ABAKI_c29400	1271.8	7194.1	4080.4	7029.8
		ABAKI_c29410	1670.5	9294.3	3278.1	4842.5

		ABAKI_c29420	935.0	4189.7	2052.7	3377.9
		ABAKI_c29430	596.9	2913.5	2087.9	3417.8
		ABAKI_c29440	1650.4	9085.0	5486.7	7648.2
Bifurcating hydrogenase	Hydrogenase (HydA1, B, D, E, C)	ABAKI_c05970	3309.5	45926.2	45994.3	157366.6
		ABAKI_c05980	4394.3	45093.6	39643.9	148985.9
		ABAKI_c05990	583.7	4143.3	5259.6	18073.5
		ABAKI_c06000	2293.8	15134.1	22079.4	49471.5
		ABAKI_c06010	1587.1	7423.0	16476.9	44070.8
