# Supplementary material for: Genome-scale analysis of Acetobacterium bakii reveals the cold adaptation of psychrotolerant acetogens by post-transcriptional regulation
Source: RNA. 2018 Dec;24(12):1839–55. doi: 10.1261/rna.068239.118 (PMC6239172; doi:10.1261/rna.068239.118)
Supplement: Supplemental Material [file supp_068239.118_Supplemental_Table_S8.pdf]

Table S8. The list of predicted non-coding RNAs.

Scaffold	Start	End	Strand	Locus_tag	TSS_ID	Rfam-accession	TSS category	Description	Type
Scaffold1	98394	98587	-	ABAKI_n00010	-	RF01482	-	AdoCbl_riboswitch	riboswitch
Scaffold1	98422	98599	-	ABAKI_n00020	-	RF00174	-	Cobalamin	riboswitch
Scaffold1	170401	170469	+	ABAKI_n00030	ABK_TSS0069	RF00559	P	L21_leader	riboswitch
Scaffold1	181495	181712	+	ABAKI_n00040	ABK_TSS0073	RF00230	N	T-box	riboswitch
Scaffold1	184065	184306	+	ABAKI_n00050	ABK_TSS0074	RF00230	N	T-box	riboswitch
Scaffold1	187205	187329	+	ABAKI_n00060	ABK_TSS0075	RF00558	P	L20_leader	riboswitch
Scaffold1	191557	191777	+	ABAKI_n00070	-	RF00230	-	T-box	riboswitch
Scaffold1	284243	284328	-	ABAKI_n00080	-	RF01051	-	c-di-GMP-I	riboswitch
Scaffold1	284409	284491	-	ABAKI_n00090	ABK_TSS0724	RF01051	N	c-di-GMP-I	riboswitch
Scaffold1	308153	308325	+	ABAKI_n00100	ABK_TSS0084	RF00168	P	Lysine	riboswitch
Scaffold1	323578	323730	-	ABAKI_n00110	-	RF00234	-	glmS	riboswitch/ribozyme
Scaffold1	371991	372185	+	ABAKI_n00120	ABK_TSS0090	-	N	Hypothetical	-
Scaffold1	371855	372194	-	ABAKI_n00130	ABK_TSS0750	RF00010	N	RNaseP_bact_a	ribozyme
Scaffold1	457457	458047	-	ABAKI_n00140	ABK_TSS0767	RF01071	N	OLE	ribozyme
Scaffold1	472846	472997	+	ABAKI_n00150	ABK_TSS0096	-	N	Hypothetical	-
Scaffold1	492663	492811	+	ABAKI_n00160	ABK_TSS0097	-	N	Hypothetical	-
Scaffold1	556691	556783	-	ABAKI_n00170	-	RF01051	-	c-di-GMP-I	riboswitch
Scaffold1	942181	942288	-	ABAKI_n00180	ABK_TSS0893	RF00059	P	TPP	riboswitch
Scaffold1	973508	973638	-	ABAKI_n00190	ABK_TSS0900	RF01055	P	MOCO_RNA_motif	riboswitch
Scaffold1	978049	978157	-	ABAKI_n00200	-	RF00059	-	TPP	riboswitch
Scaffold1	1022364	1022839	-	ABAKI_n00210	ABK_TSS0914	-	N	Hypothetical	-
Scaffold1	1045087	1045202	-	ABAKI_n00220	ABK_TSS0919	RF01055	N	MOCO_RNA_motif	riboswitch
Scaffold1	1049263	1049365	-	ABAKI_n00230	ABK_TSS0922	RF00059	P	TPP	riboswitch
Scaffold1	1085917	1087068	-	ABAKI_n00240	ABK_TSS0936	-	A	Hypothetical	-
Scaffold1	1206292	1206689	-	ABAKI_n00250	ABK_TSS0944	-	N	Hypothetical	-
Scaffold1	1319168	1319260	+	ABAKI_n00260	ABK_TSS0139	RF01831	P	THF	riboswitch
Scaffold1	1325627	1326010	-	ABAKI_n00270	ABK_TSS0963	-	N	Hypothetical	-
Scaffold1	1328832	1328924	+	ABAKI_n00280	ABK_TSS0141	RF01831	P	THF	riboswitch
Scaffold1	1335192	1335318	-	ABAKI_n00290	ABK_TSS0965	RF00557	P	L10_leader	riboswitch
Scaffold1	1413288	1413515	-	ABAKI_n00300	ABK_TSS0995	RF00230	N	T-box	riboswitch
Scaffold1	1494422	1494567	+	ABAKI_n00310	-	RF02391	-	sau-50	small RNA

Scaffold1	1540639	1540819	-	ABAKI_n00320	ABK_TSS1041	RF00174	N	Cobalamin	riboswitch
Scaffold1	1568244	1568442	-	ABAKI_n00330	-	RF00174	-	Cobalamin	riboswitch
Scaffold1	1572439	1572634	-	ABAKI_n00340	ABK_TSS1047	RF00174	N	Cobalamin	riboswitch
Scaffold1	1573090	1573275	-	ABAKI_n00350	ABK_TSS1048	RF00174	N	Cobalamin	riboswitch
Scaffold1	1580743	1581035	-	ABAKI_n00360	ABK_TSS1051	-	N	Hypothetical	-
Scaffold1	1601055	1601403	-	ABAKI_n00370	ABK_TSS1058	-	N	Hypothetical	-
Scaffold1	1608288	1608486	-	ABAKI_n00380	-	RF00174	-	Cobalamin	riboswitch
Scaffold1	1609806	1609981	-	ABAKI_n00390	ABK_TSS1059	RF00174	N	Cobalamin	riboswitch
Scaffold1	1631014	1631212	-	ABAKI_n00400	-	RF00174	-	Cobalamin	riboswitch
Scaffold1	1648237	1648303	-	ABAKI_n00410	-	RF00174	-	Cobalamin	riboswitch
Scaffold1	1692462	1693051	-	ABAKI_n00420	ABK_TSS1071	-	A	Hypothetical	-
Scaffold1	1759983	1760361	-	ABAKI_n00430	ABK_TSS1084	RF00230	N	T-box	riboswitch
Scaffold1	1813245	1813373	-	ABAKI_n00440	ABK_TSS1098	RF00050	P	FMN	riboswitch
Scaffold1	1814556	1814719	-	ABAKI_n00450	ABK_TSS1099	RF00380	P	ykoK	riboswitch
Scaffold1	1827890	1828153	-	ABAKI_n00460	ABK_TSS1105	RF00230	N	T-box	riboswitch
Scaffold1	1971876	1971969	-	ABAKI_n00470	ABK_TSS1135	RF01051	P	c-di-GMP-I	riboswitch
Scaffold1	2044851	2044910	+	ABAKI_n00480	ABK_TSS0177	RF01734	P	crcB	riboswitch
Scaffold1	2206775	2206859	+	ABAKI_n00490	ABK_TSS0217	RF01051	N	c-di-GMP-I	riboswitch
Scaffold1	2308461	2308577	+	ABAKI_n00500	ABK_TSS0238	RF00050	P	FMN	riboswitch
Scaffold1	2373165	2373385	-	ABAKI_n00510	ABK_TSS1194	-	N	Hypothetical	-
Scaffold1	2376697	2376962	+	ABAKI_n00520	ABK_TSS0253	RF01854	N	Bacteria_large_SRP	srp
Scaffold1	2376811	2376910	+	ABAKI_n00530	ABK_TSS1195	RF00169	N	Bacteria_small_SRP	srp
Scaffold1	2383864	2384045	+	ABAKI_n00540	ABK_TSS0257	-	N	Hypothetical	-
Scaffold1	2389915	2390217	+	ABAKI_n00550	ABK_TSS0260	-	A	Hypothetical	-
Scaffold1	2399915	2400502	+	ABAKI_n00560	ABK_TSS0266	-	N	Hypothetical	-
Scaffold1	2422297	2422556	+	ABAKI_n00570	ABK_TSS0272	-	N	Hypothetical	-
Scaffold1	2459067	2459156	+	ABAKI_n00580	ABK_TSS0277	RF01051	P	c-di-GMP-I	riboswitch
Scaffold1	2517875	2518126	+	ABAKI_n00590	ABK_TSS0290	-	N	Hypothetical	-
Scaffold1	2594277	2594486	+	ABAKI_n00600	ABK_TSS0311	RF00230	N	T-box	riboswitch
Scaffold1	2604875	2605047	+	ABAKI_n00610	-	RF02001	-	group-II-D1D4-3	ribozyme
Scaffold1	2606930	2607381	+	ABAKI_n00620	ABK_TSS0316	-	N	Hypothetical	-
Scaffold1	2680742	2681050	+	ABAKI_n00630	ABK_TSS0328	-	N	Hypothetical	-
Scaffold1	2699449	2699703	+	ABAKI_n00640	ABK_TSS0335	-	N	Hypothetical	-
Scaffold1	2787126	2787419	+	ABAKI_n00650	ABK_TSS0363	-	N	Hypothetical	-

Scaffold1	2796117	2796213	+	ABAKI_n00660	ABK_TSS0369	RF00162	P	SAM	riboswitch
Scaffold1	2853547	2854201	+	ABAKI_n00670	ABK_TSS0379	-	A	Hypothetical	-
Scaffold1	2855978	2856075	+	ABAKI_n00680	ABK_TSS0381	RF00162	P	SAM	riboswitch
Scaffold1	2881526	2881736	-	ABAKI_n00690	ABK_TSS1243	RF00230	P	T-box	riboswitch
Scaffold1	2911743	2911818	+	ABAKI_n00700	-	RF01051	-	c-di-GMP-I	riboswitch
Scaffold1	2965582	2965698	-	ABAKI_n00710	ABK_TSS1250	RF01055	P	MOCO_RNA_motif	riboswitch
Scaffold1	3039275	3039455	-	ABAKI_n00720	-	RF00174	-	Cobalamin	riboswitch
Scaffold1	3049235	3049431	+	ABAKI_n00730	ABK_TSS0437	-	N	Hypothetical	-
Scaffold1	3159959	3160665	+	ABAKI_n00740	ABK_TSS0456	-	A	Hypothetical	-
Scaffold1	3166586	3166711	-	ABAKI_n00750	ABK_TSS1264	RF01055	N	MOCO_RNA_motif	riboswitch
Scaffold1	3295680	3295934	+	ABAKI_n00760	ABK_TSS0494	RF00230	N	T-box	riboswitch
Scaffold1	3406914	3407809	-	ABAKI_n00770	ABK_TSS1295	-	N	Hypothetical	-
Scaffold1	3669946	3670531	+	ABAKI_n00780	ABK_TSS0571	-	N	Hypothetical	-
Scaffold1	3763263	3763663	+	ABAKI_n00790	-	RF00023	-	tmRNA	tmRNA
Scaffold1	3773632	3774003	+	ABAKI_n00800	-	RF00023	-	tmRNA	tmRNA
Scaffold1	3792017	3792117	+	ABAKI_n00810	ABK_TSS0601	RF01764	P	yjdF	riboswitch
Scaffold1	3821896	3822084	+	ABAKI_n00820	-	RF00174	-	Cobalamin	riboswitch
Scaffold1	3827617	3827717	+	ABAKI_n00830	ABK_TSS0606	RF01764	P	yjdF	riboswitch
Scaffold1	3833823	3834513	+	ABAKI_n00840	ABK_TSS0609	-	N	Hypothetical	-
Scaffold1	3852169	3852258	+	ABAKI_n00850	-	RF01749	-	pan	riboswitch
Scaffold1	3878451	3879467	-	ABAKI_n00860	ABK_TSS1337	-	A	Hypothetical	-
Scaffold1	3937178	3937925	+	ABAKI_n00870	ABK_TSS0633	-	N	Hypothetical	-
Scaffold1	4255280	4255452	+	ABAKI_n00880	ABK_TSS0677	RF00174	N	Cobalamin	riboswitch
