# Supplementary material for: Genome-scale analysis of Acetobacterium bakii reveals the cold adaptation of psychrotolerant acetogens by post-transcriptional regulation
Source: RNA. 2018 Dec;24(12):1839–55. doi: 10.1261/rna.068239.118 (PMC6239172; doi:10.1261/rna.068239.118)
Supplement: Supplemental Material [file supp_068239.118_Supplemental_Table_S9.pdf]

Table S9. Attenuators identified in *A. bakii* and their respective mRNAs.

Locus_Tag	TSS_ID	Rfam-accession #	Description	CDS	Function	DEG cluster
ABAKI_n00760	ABK_TSS0494	RF00230	T-box	ABAKI_c30200	Anthranilate synthase component I	C1
ABAKI_n00090	ABK_TSS0724	RF01051	c-di-GMP-I	ABAKI_c02730	Peptidase A24 family	C3
ABAKI_n00810	ABK_TSS0601	RF01764	yjdF	ABAKI_c34680	Uncharacterized protein	C3
ABAKI_n00030	ABK_TSS0069	RF00559	L21_leader	ABAKI_c01690	50S ribosomal protein L21	C4
ABAKI_n00260	ABK_TSS0139	RF01831	THF	ABAKI_c12740	Uncharacterized protein	C4
ABAKI_n00470	ABK_TSS1135	RF01051	c-di-GMP-I	ABAKI_c18600	Uncharacterized protein	C4
ABAKI_n00480	ABK_TSS0177	RF01734	crcB	ABAKI_c19170	Putative fluoride ion transporter CrcB	C4
ABAKI_n00440	ABK_TSS1098	RF00050	FMN	ABAKI_c17190	Riboflavin transporter	C5
ABAKI_n00500	ABK_TSS0238	RF00050	FMN	ABAKI_c21530	Riboflavin biosynthesis protein RibD	C5
ABAKI_n00040	ABK_TSS0073	RF00230	T-box	ABAKI_c01830	Threonine--tRNA ligase	C7
ABAKI_n00280	ABK_TSS0141	RF01831	THF	ABAKI_c12850	GTP cyclohydrolase 1	C7
ABAKI_n00290	ABK_TSS0965	RF00557	L10_leader	ABAKI_c12920	50S ribosomal protein L10	C7
ABAKI_n00340	ABK_TSS1047	RF00174	Cobalamin	ABAKI_c15170	Precorrin-6A reductase CbiJ1	C7
ABAKI_n00350	ABK_TSS1048	RF00174	Cobalamin	ABAKI_c15170	Precorrin-6A reductase CbiJ1	C7
ABAKI_n00680	ABK_TSS0381	RF00162	SAM	ABAKI_c26320	S-adenosylmethionine synthase	C7
ABAKI_n00690	ABK_TSS1243	RF00230	T-box	ABAKI_c26520	Glycine--tRNA ligase	C7
ABAKI_n00220	ABK_TSS0919	RF01055	MOCO_RNA_motif	ABAKI_c09940	Aldehyde oxidase and xanthine dehydrogenase molybdopterin binding protein	C9
ABAKI_n00060	ABK_TSS0075	RF00558	L20_leader	ABAKI_c01870	Translation initiation factor IF-3	C10
ABAKI_n00580	ABK_TSS0277	RF01051	c-di-GMP-I	ABAKI_c22940	Uncharacterized protein	C10
ABAKI_n00050	ABK_TSS0074	RF00230	T-box	ABAKI_c01840	ABC transport system substrate-binding protein	C11
ABAKI_n00180	ABK_TSS0893	RF00059	TPP	ABAKI_c08910	Uncharacterized protein	C11
ABAKI_n00230	ABK_TSS0922	RF00059	TPP	ABAKI_c09970	Phosphomethylpyrimidine synthase	C11
ABAKI_n00320	ABK_TSS1041	RF00174	Cobalamin	ABAKI_c14930	Cobalt transport protein CbiM	C11
ABAKI_n00430	ABK_TSS1084	RF00230	T-box	ABAKI_c16750	2-isopropylmalate synthase LeuA1	C11
ABAKI_n00660	ABK_TSS0369	RF00162	SAM	ABAKI_c25820	Methionine synthase MetH	C11
ABAKI_n00100	ABK_TSS0084	RF00168	Lysine	ABAKI_c02960	Aspartate-semialdehyde dehydrogenase	N/A
ABAKI_n00190	ABK_TSS0900	RF01055	MOCO_RNA_motif	ABAKI_c09210	Molybdopterin biosynthesis protein MoeA1	N/A
ABAKI_n00300	ABK_TSS0995	RF00230	T-box	ABAKI_c13650	Alanine--tRNA ligase	N/A
ABAKI_n00390	ABK_TSS1059	RF00174	Cobalamin	ABAKI_c15480	Uncharacterized protein	N/A
ABAKI_n00450	ABK_TSS1099	RF00380	ykoK	ABAKI_c17210	Uncharacterized protein	N/A
ABAKI_n00460	ABK_TSS1105	RF00230	T-box	ABAKI_c17310	Phenylacetate-coenzyme A ligase	N/A
ABAKI_n00490	ABK_TSS0217	RF01051	c-di-GMP-I	ABAKI_c20560	Uncharacterized protein	N/A
ABAKI_n00600	ABK_TSS0311	RF00230	T-box	ABAKI_c24090	Isoleucine--tRNA ligase	N/A
ABAKI_n00710	ABK_TSS1250	RF01055	MOCO_RNA_motif	ABAKI_c27150	Extracellular tungstate binding protein	N/A
ABAKI_n00750	ABK_TSS1264	RF01055	MOCO_RNA_motif	ABAKI_c29050	Molybdopterin converting factor, subunit 1	N/A

ABAKI_n00830	ABK_TSS0606	RF01764	yjdF	ABAKI_c34990	Uncharacterized protein	N/A
ABAKI_n00880	ABK_TSS0677	RF00174	Cobalamin	ABAKI_c39140	ATPase components of various ABC-type transport systems, containing duplicated ATPase	N/A
