# Supplementary material for: Genome-scale analysis of Acetobacterium bakii reveals the cold adaptation of psychrotolerant acetogens by post-transcriptional regulation
Source: RNA. 2018 Dec;24(12):1839–55. doi: 10.1261/rna.068239.118 (PMC6239172; doi:10.1261/rna.068239.118)
Supplement: Supplemental Material [file supp_068239.118_Supplemental_Table_S10.pdf]

Table S10. Oligonucleotides and DNA fragments used in this study. The list of DNA and RNA oligonucleotides for dRNA-seq library preparation, qRT-PCR, and 5' RACE analysis. RED: Illumina Tru-seq Index; Index 1, 2, 3, 4, 5, 6, 7, 8, 9, 10, 11, 12, 13, 19, 22, and 27 were used in this study.

Methods	name	Length (nt)	Sequence	Used strain	Target gene	Function
**dRNA-seq**	5' RNA adaptor	33	ACACUCUUUCCCUACACGACGCUCUU CCGAUCU	*A. bakii*	N/A	N/A
	Solexa_Mul_RT_random primer_N9 (3' random primer)	43	GTGACTGGAGTTCAGACGTGTGCTCTT CCGATCT N9	*A. bakii*	N/A	N/A
	Solexa_Mul_PCR_1 (5' PCR universal primer)	58	AATGATACGGCGACCACCGAGATCTAC ACTCTTTCCCTACACGACGCTCTTCCGA TCT	*A. bakii*	N/A	N/A
	Solexa_Mul_ln_XX_long (Index primer)	64-66	CAAGCAGAAGACGGCATACGAGATXX XXXXGTGACTGGAGTTCAGACGTGTGC TCTTCCGATCT	*A. bakii*	N/A	N/A
**qRT-PCR**	BA_qPCR_secA_F	20	GGATAACATGGCCCCCACAA	*A. bakii*	ABAKI_c25770	Protein translocase subunit SecA
	BA_qPCR_secA_R	20	AAGGGGTTCTGGCTTCATCG	*A. bakii*		
	BA_qPCR_HydA_F	20	TGCTTTGCATTTCTGGACGC	*A. bakii*	ABAKI_c09070	Iron hydrogenase HydA2
	BA_qPCR_HydA_R	20	ATGTCCAGTGTCTGCCATCG	*A. bakii*		
	BA_qPCR_acsD_F	20	TGCAACGCAGTTTTCCACTG	*A. bakii*	ABAKI_c13120	Corrinoid/iron-sulfur protein, small subunit AcsD
	BA_qPCR_acsD_R	20	CTTGGGCAAAAGCAGCAGTT	*A. bakii*		
	BA_qPCR_metF_F	20	GTACCGGTTCACTGGCAACT	*A. bakii*	ABAKI_c24840	Methylenetetrahydrofolate reductase large subunit MetF
	BA_qPCR_metF_R	20	ATCGGACTGCAGGGCAATAC	*A. bakii*		
	BA_qPCR_lctB_F	20	ATGATTCGCGATGGGGTTGA	*A. bakii*	ABAKI_c24310	Electron transfer flavoprotein beta-subunit EtfB
	BA_qPCR_lctB_R	20	CTGTAATGGTTCCGCCCAGT	*A. bakii*		
	BA_qPCR_pyc_F	20	ACCAACTCGGTGACCGTATG	*A. bakii*	ABAKI_c07540	Pyruvate carboxylase
	BA_qPCR_pyc_R	20	ACGCCGGTACCATTGAGTTT	*A. bakii*		
	WO_qPCR_secA_F	20	GGAGCTTGACAGCCGTTTTG	*A. woodii*	Awo_c28060	Protein translocase subunit SecA
	WO_qPCR_secA_R	20	CAAGGGTTTCTCCTTGCGCT	*A. woodii*		
	WO_qPCR_HydA_F	20	AATGTGCCGAAGTGTGTCCA	*A. woodii*	Awo_c08260	Iron hydrogenase
	WO_qPCR_HydA_R	20	ATTTCTGTACGCATTGGCCG	*A. woodii*		
	WO_qPCR_acsD_F	20	TTGGGCAGAGTGCTTTGGAA	*A. woodii*	Awo_c10710	Corrinoid/iron-sulfur protein, small subunit AcsD
	WO_qPCR_acsD_R	21	ACAGCAAGAAATCAGCACCTG	*A. woodii*		

	WO_qPCR_metF_F	20	TGCGTACTGGTTCATTGGCA	*A. woodii*	Awo_c09310	Methylenetetrahydrofolate reductase large subunit MetF
	WO_qPCR_metF_R	21	GGCAATACGATTTCGATCCCG	*A. woodii*		
	WO_qPCR_lctB_F	20	AGAACAATTAGGCGGGACCA	*A. woodii*	Awo_c08710	Electron transfer flavoprotein beta-subunit EtfB
	WO_qPCR_lctB_R	20	CCTCATCAGCGCCCATGTAA	*A. woodii*		
**5'RACE**	FW.5bt (Short RNA adapter)	15	ACGGACUAGAAGAAA	*A. bakii*	N/A	N/A
	FR.RNA5 (Second RNA adapter)	38	AUAUGCGCGAAUUCCUGUAGAACGAACACUAGAAGAAA	*A. bakii*	N/A	N/A
	FR.DNA5	20	GCGCGAATTCCTGTAGAACG	*A. bakii*	N/A	N/A
	BA_5RACE_fdhF2_R	20	TGGGGTCGCGCTAACAATTT	*A. bakii*	ABAKI_c09110	Formate dehydrogenase H
	BA_5RACE_acsD_R	20	TGTTTTCGCCCCCTATGCTT	*A. bakii*	ABAKI_c13120	Corrinoid/iron-sulfur protein, small subunit AcsD
	BA_5RACE_cooC1_R	20	CCCAGACCTTCTGCAACCAA	*A. bakii*	ABAKI_c13160	CO dehydrogenase nickel-insertion accessory protein CooC1
	BA_5RACE_2680710N_R	21	TCACCACCTTCTTTCTTGCGA	*A. bakii*	N/A	N/A
	BA_5RACE_fhs1_R	23	TCTGTTAAACCCAACTTAGCTGC	*A. bakii*	ABAKI_c24790	Formate--tetrahydrofolate ligase
	BA_5RACE_rnfC1_R	20	TGGCACATCGGCTGTACTTT	*A. bakii*	ABAKI_c29390	Electron transport complex subunit C
**Assays for p5'UTR-GFP reporter constructs**	pHCE_EGFP-rrnBT_F	34	TACCGAGCTCGAATTCCCATGGTGAGCAAGGGCGA	*E. coli*	N/A	N/A
	pHCE_EGFP-rrnBT_R	38	TGAGAGTGCACCATAATTTGTCCTACTCAGGAGAGCGT	*E. coli*	N/A	N/A
	pTRC + cspA-5'UTR	229	CGACTCTAGAGGATCCTTGACAATTAATCATCCGGctcgtataatgtgtggaattacggtttgacgtacagaccattaaagcagtgtagtaaggcaagtcccttcaagagttatcgttgatacccctcgtagtgcacattcctttaacgcttcaaaatctgtaaagcacgccatatcgccgaaaggcacaCTTAATTATTAAAGGTAATACACTCATGGTGAGCAAGGG	*E. coli*	N/A	N/A

	pTRC + cooC1-5’UTR	190	CGACTCTAGAGGATCCTTGACAATTAATCATCCGGCtcgtataatgtgtggaattCGATACATATATATACAAGTTGATAAGAAAACAAAATTTTCCTGTTGTAAGTTCATATGTTATAACTTAAACTGGATTATAACAAATTAATGCGAAAAAATTATTTTGGAGGAAAAAATTCATGGTGAGCAAGGG	*E. coli*	N/A	N/A
	pTRC + fhs1-5’UTR	118	CGACTCTAGAGGATCCTTGACAATTAATCATCCGGCTCGtataatgtgtggaattAGATGTTAAAAATTTATCAAACTAAATGGAGGAAATTTTATGGGTTTTCATGGTGAGCAAGGG	*E. coli*	N/A	N/A
	pTRC + Ef-P-5’UTR	113	CGACTCTAGAGGATCCTTGACAATTAATCATCCGGctcgtataatgtgtggaattATTATACCTCCGCTATGGGTTTGTTTTCTATCTATAAATATTTCATGGTGAGCAAGGG	*E. coli*	N/A	N/A
	qPCR_GFP_F	20	CTACCCCGACCACATGAAGC	*E. coli*	eGFP	eGFP
	qPCR_GFP_R	20	CTTGTAGTTGCCGTCGTCCT	*E. coli*		
	qPCR_gyrA_F	20	ACGCGACTTGGTTGGGTATT	*E. coli*	EG10423 (EcoCyc)	DNA gyrase subunit A
	qPCR_gyrA_R	20	GTCTCTCTGATCGTGCCTCG	*E. coli*		
